# nanoRAPIDS as an analytical pipeline for the discovery of novel bioactive metabolites in complex culture extracts at the nanoscale

**DOI:** 10.1038/s42004-024-01153-y

**Published:** 2024-04-01

**Authors:** Isabel Nuñez Santiago, Nataliia V. Machushynets, Marija Mladic, Doris A. van Bergeijk, Somayah S. Elsayed, Thomas Hankemeier, Gilles P. van Wezel

**Affiliations:** 1 Molecular Biotechnology, Institute of Biology, Leiden University, Leiden, The Netherlands; 2 DSM-Firmenich, Delft, The Netherlands; 3 Department of Microbiology, KU Leuven, Immunology and Transplantation (Laboratory of Molecular Bacteriology), Leuven, Belgium; 4 VIB, Center for Microbiology, Leuven, Belgium; 5 Leiden Academic Centre for Drug Research (LACDR), Leiden University, Leiden, The Netherlands

**Keywords:** Natural product synthesis, Microbiology, Mass spectrometry

## Abstract

Microbial natural products form the basis of most of the antibiotics used in the clinic. The vast majority has not yet been discovered, among others because the hidden chemical space is obscured by previously identified (and typically abundant) antibiotics in culture extracts. Efficient dereplication is therefore key to the discovery of our future medicines. Here we present an analytical platform for the efficient identification and prioritization of low abundance bioactive compounds at nanoliter scale, called nanoRAPIDS. NanoRAPIDS encompasses analytical scale separation and nanofractionation of natural extracts, followed by the bioassay of interest, automated mass spectrometry identification, and Global Natural Products Social molecular networking (GNPS) for dereplication. As little as 10 μL crude extract is fractionated into 384 fractions. First, bioactive congeners of iturins and surfactins were identified in *Bacillus*, based on their bioactivity. Subsequently, bioactive molecules were identified in an extensive network of angucyclines elicited by catechol in cultures of *Streptomyces* sp. This allowed the discovery of a highly unusual N-acetylcysteine conjugate of saquayamycin, despite low production levels in an otherwise abundant molecular family. These data underline the utility and broad application of the technology for the prioritization of minor bioactive compounds in complex extracts.

## Abstract (web summary)

Microbial natural products are an important source for antibiotic discovery, however, their efficient dereplication remains challenging. Here, the authors develop an analytical pipeline, nanoRAPIDS, to prioritize low abundance bioactive compounds at nanoscale, by integrating the bioassay of interest, automated mass spectrometry identification and GNPS-based dereplication, resulting in the discovery of saquayamycin N from *Streptomyces* sp. MBT84.

## Introduction

The increasing number of antibiotic resistant bacteria combined with the decreasing number of new antibiotics discovered is becoming a serious threat to public health. The traditional source of antibiotics is natural products produced by microorganisms, whereby some 70% are produced by Actinobacteria^1,2^. The canonical antibiotic discovery approach, known as the “Waksman platform”^3^, involves high-throughput screening of Actinobacteria for antimicrobial activity, but while this approach was highly successful in the golden era of antibiotic discovery, nowadays that approach fails to identify truly novel classes of compounds^4^. Still, we have likely only exploited a fraction of the chemical space of microbial natural products^5^. Indeed, the molecules that are currently described^6^ likely only represent 3% of the extant chemical space of natural products^7^. The challenge lies in the development of technologies to access the undiscovered chemical space of natural products. Genome sequencing revealed that *Streptomyces* genomes each contain up to 60 or even more biosynthetic gene clusters (BGCs) that specify natural products^8^. Bioinformatic tools such as antiSMASH^9^ allow visualizing these BGCs and predicting the classes of molecules they produce. However, many of these BGCs are expressed at a very low level under routine laboratory conditions, and hence their cognate products cannot be elucidated. Actinobacteria are usually grown in isolation and in laboratory media that do not accurately mimic the complexity of their natural habitat. The control of BGCs is most likely tightly tied to the competitive ecological conditions in which antibiotic production evolved^10,11^.

Several methods have been developed to boost the production of hitherto hidden natural products, including ribosome engineering, co-cultivation, OSMAC (One strain-many compounds), promoter replacement, and high-throughput elicitor screening (reviewed in refs. ^12,13^). Metabolites are identified by liquid chromatography coupled to mass spectrometry (LC–MS) and NMR spectroscopy. Statistical models such as Pearson correlation, partial least squares (PLS), discriminant analysis (PCA-DA, PLS-DA, OPLS-DA), and hierarchical cluster analysis (HCA) are then used to link metabolite fingerprints to bioactivity data^14^. Identifying the bioactive compounds of interest is often time-consuming and expensive, while efficient dereplication is essential to avoid wasting time and resources on the rediscovery of known compounds. Global Natural Products Social (GNPS) molecular networking developed in the Dorrestein laboratory^15^ is an important addition to the toolbox. Although these approaches provide lists of highlighted features, the information obtained is not immediately linked to bioactivity. Several analytical modules involving different bioassays and detection technologies can be linked to allow simultaneous bioactivity evaluation and identification of compounds present in small amounts (analytical scale) in complex compound mixtures. Such approaches include the at-line high-resolution nanofractionation, which could complement previously mentioned strategies and provide a more comprehensive insight into compounds responsible for the activity^16,17^.

One of the major challenges is the lack of truly novel chemical scaffolds, which may serve as the basis for the antibiotics of tomorrow. We discovered lugdunomycin that is produced by the soil-derived *Streptomyces* sp. QL37^18^. Lugdunomycin is a highly rearranged member of the angucyclines, the largest family of type II polyketides^19,20^, and has a radically different chemical scaffold as compared to other polyketides. Its discovery underlines that novel chemical space may still lie hidden even in highly explored chemical families.

To allow more efficient mining of microbial extracts for novel chemical space, we developed an analytical platform for the rapid and efficient identification and dereplication of bioactive metabolites based on nanofractionation (nanoRAPIDS). NanoRAPIDS combines at-line high-resolution nanofractionation^21^ coupled to LC–MS/MS and featured-based molecular networking (FBMN)^22^ in a single analysis. The implementation of MZmine^23^ in our platform allows for automatic peak selection and correlation of the bioactivity with the appropriate mass while the same workflow performs FBMN. This allows dereplication and analog searches in a semi-quantitative fashion. The nanoRAPIDS workflow was validated by identifying a suite of iturins and surfactins produced by *Bacillus* sp. 90A-23 based on their bioactivity against *Escherichia coli*, *Bacillus subtilis*, and/or *Aspergillus niger* in nanoliter fractions. We then applied the technology to prioritize bioactive angucyclines produced by *Streptomyces* sp. MBT84 following challenge by catechol. This afforded the isolation of a hitherto unknown N-acetylcysteine conjugate of saquayamycin N.

## Results and discussion

### Development of the nanoRAPIDS platform

**Fig. 1:**
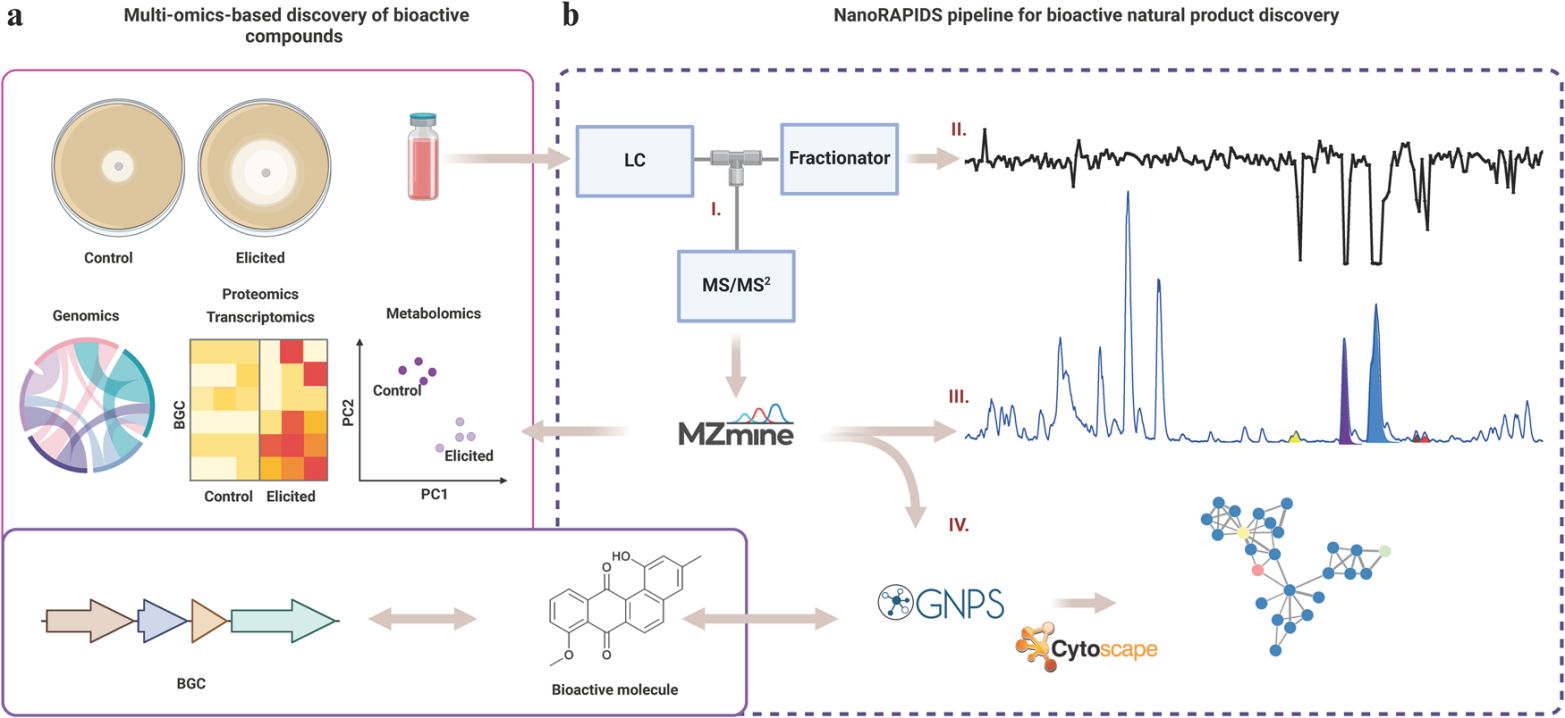
Schematic overview of the nanoRAPIDS platform. **a** Classical omics-based pipeline for the discovery of bioactive compounds. Multi-omics approaches advance the field of microbial natural products research. However, despite significant improvements in metabolite profiling, the unambiguous identification of each individual NP in an extract using generic methods remains challenging. **b** Integration of the nanoRAPIDS pipeline into the multi-omics workflow allows for the detection and dereplication of the compounds contributing to the bioactivity of the complex extract. The nanoRAPIDS pipeline starts with at-line nanofractionation (I) and parallel MS/MS^2^, followed by bioactivity assays of the individual nanofractions through the resazurin reduction assay (II) to determine their retention time (RT). The MS/MS^2^ data are automatically pre-processed in MZmine to determine the *m/z* values of the bioactive features (III). The peak list is then subjected to GNPS molecular networking and dereplication. Molecular network visualization and automatic bioactive feature mapping are conducted in Cytoscape (IV).

The challenge we sought to overcome is that bioactivity-guided fractionation is typically frustrated by highly abundant known antibiotics, which obscures bioactive compounds that are produced at low levels. This is particularly true for screening of small culture volumes, such as those obtained through HT cultivation. Therefore, we developed an antibiotic discovery platform based on nanofractionation^16^, mass spectrometry, and bioactivity assays. This set-up was combined with automated data pre-processing in MZmine 2.53^23^ and feature-based molecular networking^22^. The pipeline is designated nanoRAPIDS, for a Reliable Analytical Platform for Identification and Dereplication of Specialized metabolites based on nanofractionation. The schematic overview of the proposed workflow is shown in Fig. 1.

The nanoRAPIDS pipeline makes use of an at-line nanofractionation set-up^21^. First, extracts are separated by liquid chromatography (LC) and after the post-column split, the largest part of the flow is fractionated using high-resolution nanofractionation, while a small part is subjected to mass spectrometry (MS and MS/MS), allowing the parallel data collection of all fractions. Next, the individual fractions are tested against target microorganisms such as Gram-positive, Gram-negative bacteria, and/or fungi with a resazurin reduction assay. Since the at-line nanofractionation is performed at a 6 s resolution, the retention time of eluting compounds from the LC separation is retained in the bioactivity chromatograms. After at-line nanofractionation, the LC-MS/MS spectral data are automatically processed with MZmine^23^, which gives the information on the *m/z* values of the bioactive fraction(s) constituents and their MS/MS spectra. Pre-processed MS^2^ data are subsequently uploaded onto the GNPS web-platform^15^ for feature-based molecular networking^22^. Molecular networking is then performed to visualize all ions of molecules detected and fragmented during an MS experiment and identify the chemical relationships between them in a semi-quantitative fashion. Additionally, GNPS automatically performs a spectral library search for known molecules in the molecular networks if their MS/MS spectra are available in public MS/MS spectral libraries. The final step of the analysis consists of mapping of the bioactive compounds within the obtained network in Cytoscape^24^, based on their *m/z* and retention time (Fig. 1b).

### Validation of nanoRAPIDS by screening *Bacillu*s sp. 90A-23 extracts

**Fig. 2:**
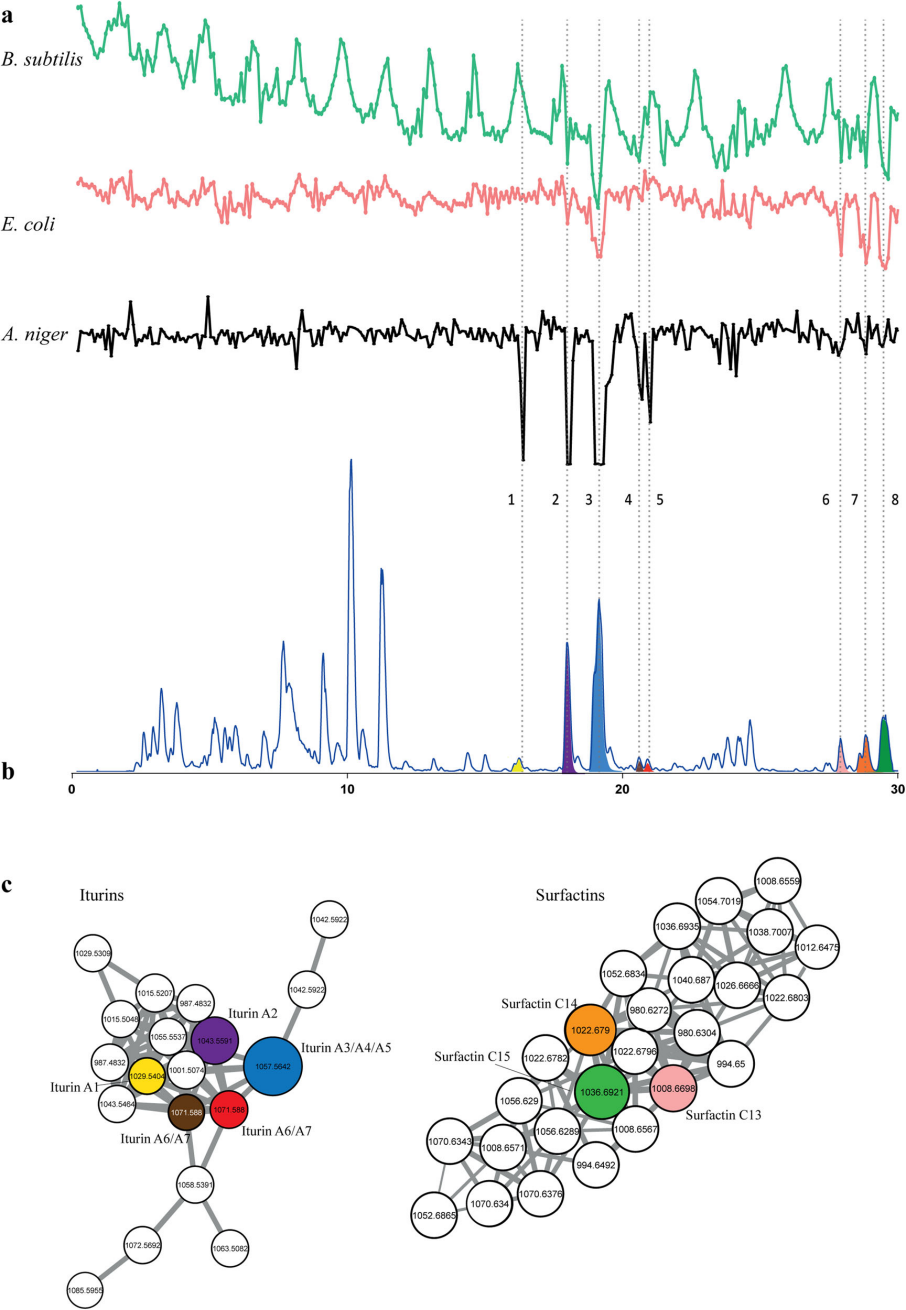
NanoRAPIDS screen of extract of *Bacillus* sp. 90A-23. **a** Chromatograms representing the results of the resazurin reduction assay against *E. coli*, *B. subtilis,* and *A. niger* for each nanofraction plotted against the time the nanofraction was collected. Fractions were collected with 6 s resolution onto 384-well plates after a 10 µL injection of crude extract obtained from *Bacillus* sp. 90A-23, at 15 mg/mL concentrations. **b** The LC–MS data presented as base peak chromatogram (BPC) with extracted ion currents of the *m/z* values found in the MS spectra corresponding to the bioactive peaks. **c** Subfamilies containing the bioactive iturins (left) and surfactins (right) as identified by GNPS-molecular networking of culture extracts. Colors of the nodes correspond to the colors of the chromatographic peaks and highlight the mass features detected in the bioactive nanofractions. The node size represents the relative abundance of ions. The edge thickness attribute is an interpretation of the cosine similarity score, with thicker lines indicating higher similarity.

*Bacillus* sp. 90A-23 produces several bioactive compounds with broad spectrum activity, many of which are well characterized, including iturins and surfactins, which both consist of families of molecules. To validate the platform, crude extracts were subjected to analysis using nanoRAPIDS (Fig. 2). Automated feature detection and correlation guided by high-resolution bioactivity assays identified eight mass features with *m/z* 1029.5404 at 16.32 min, *m/z* 1043.5591 at 18.05 min, *m/z* 1057.5642 at 19.22 min, *m/z* 1071.5880 at 20.64 min, *m/z* 1071.5880 at 20.95 min, *m/z* 1008.6698 at 27.91 min, *m/z* 1022.6782 at 28.84 min and *m/z* 1036.6921 at 29.47 min (Table 1). It is important to note that some compounds may remain undetected due to the limitation of the ionization method from the MS system.

**Table 1:** Bioactive compounds identified in extracts of *Bacillus* sp. 90A-23

Peak	Mass (*m/z*)	RT(min)	Molecular formula	Identified compound	Reference
**1**	1029.5404	16.32	C_47_H_72_O_14_N_12_	(C_13_) Iturin A1 [M + H]^+^	^27^
**2**	1043.5591	18.05	C_48_H_74_O_14_N_12_	(C_14_) Iturin A2 [M + H]^+^	^50^
**3**	1057.5642	19.22	C_49_H_76_O_14_N_12_	(C_15_) Iturin A3/A4/A5 [M + H]^+^	^27^
**4**	1071.5880	20.64	C_50_H_78_O_14_N_12_	(C_16_) Iturin A6/A7 [M + H]^+^	^51^
**5**	1071.5880	20.95	C_50_H_78_O_14_N_12_	(C_16_) Iturin A6/A7 [M + H]^+^	^51^
**6**	1008.6698	27.91	C_51_H_89_N_7_O_13_	Surfactin Leu/Ile7 C13	^27,31,52^
**7**	1022.6782	28.84	C_52_H_91_N_7_O_13_	Surfactin Leu/Ile7 C14	^27,31,52^
**8**	1036.6921	29.47	C_53_H_93_N_7_O_13_	Surfactin Leu/Ile7 C15	^27,31,52^

**Fig. 3:**
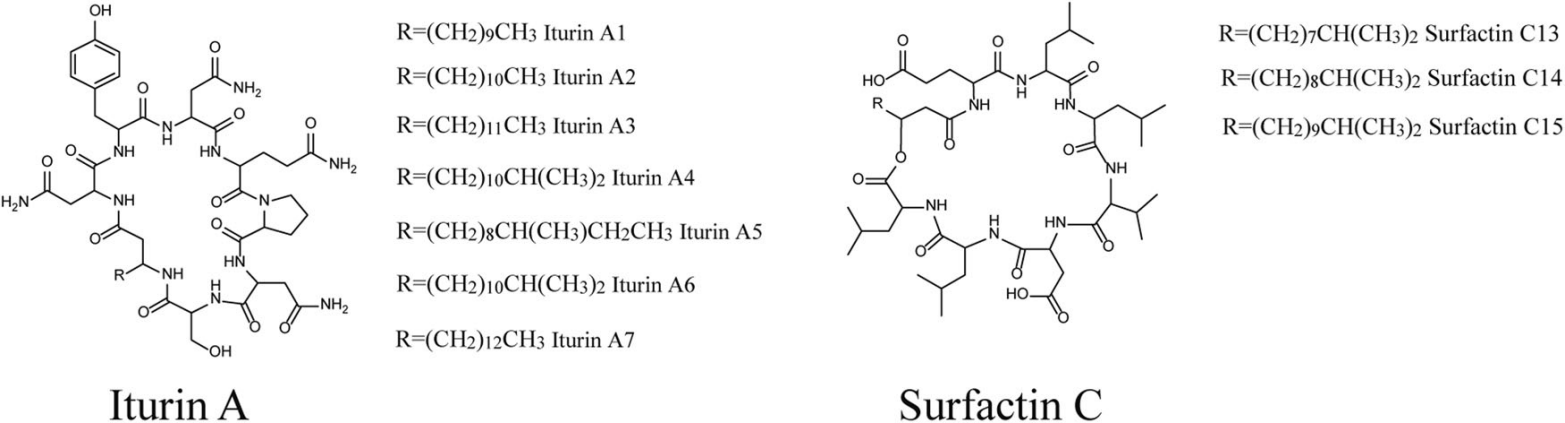
Bioactive compounds found in the extracts of *Bacillus* sp. 90A-23. Structures of iturins and surfactins detected in the extracts of *Bacillus* sp. 90A-23. R, the site where the fatty acid chain is linked to the peptidic backbone.

The filtered peak list generated in MZmine was exported for GNPS molecular networking. The network consisted of 852 nodes, which were clustered in 92 spectral families and 134 individual nodes. The bioactive compounds with *m/z* 1029.5404 at 16.32, *m/z* 1043.5591 at 18.05 min, *m/z* 1057.5642 at 19.22 min, *m/z* 1071.5880 at 20.64 min, *m/z* 1071.5880 at 20.95 min were found in a 19-node cluster (Fig. 2c). Search each MS/MS spectrum against the GNPS spectral libraries resulted in the annotation of *m/z* 1043.5591, *m/z* 1057.5642, *m/z* 1071.5880 as iturin A2, A3/4/5 and A6/7, respectively. Annotation of these compounds was supported by a direct comparison of the MS/MS spectra with the standards of iturins from GNPS by mirror plot using mass software^25^ (Figs. S2–S4). The compound with *m/z* 1029.5404 at 16.32 min was annotated as iturin A1 using the Antibase database^26^ and manual comparison of MS/MS spectra with those in the literature^27^. These amphiphilic compounds are characterized by a peptide ring of seven amino acid residues, closed by a β-amino fatty acid (Fig. 3). Iturins are a family of lipopeptides produced by various Bacilli  species^28–30^. The 14 Da difference between molecules with *m/z* 1029.5404, *m/z* 1043.5591, *m/z* 1057.5642, and *m/z* 1071.5880 is explained by variation in length of the fatty acid chain, containing 13, 14, 15 or 16 carbons, respectively (Fig. S5).

The major peaks in the bioactivity chromatogram at 18.05 and 19.22 min corresponded to the most intense compounds, which were dereplicated as iturin A2 (*m/z* 1043.5591) and iturin A3/A4 (*m/z* 1057.5642). Moreover, 12 more mass spectral features were also identified as iturins based on analog searches. The bioactive features with *m/z* 1008.6698 at 27.91 min, *m/z* 1022.6782 at 28.84 min and *m/z* 1036.6921 at 29.47 min were detected within the surfactin spectral family (Fig. 2c).

The molecule with *m/z* 1008.6698 was annotated as surfactin Leu/Ile7 C13, that with *m/z* 1022.6782 as surfactin Leu/Ile7 C14 and the compound with *m/z* 1036.6921 as surfactin Leu/Ile7 C15^31^ (Figs. S6–S9). An additional 23 other spectral features also belonged to the same spectral family.

In the initial screen, *Bacillus* sp. 90A-23 showed bioactivity against both bacteria and fungi. NanoRAPIDS allowed us to readily establish which detected individual mass features contributed to the bioactive nanofraction. The analysis revealed that the initially detected antifungal and antibacterial activity of the crude extract was mostly caused by iturins and surfactins (Fig. 2 and Table 1).

### Identification of a *N*-acetylcysteine conjugate in a complex network of angucyclines in *Streptomyces* sp. MBT84

**Fig. 4:**
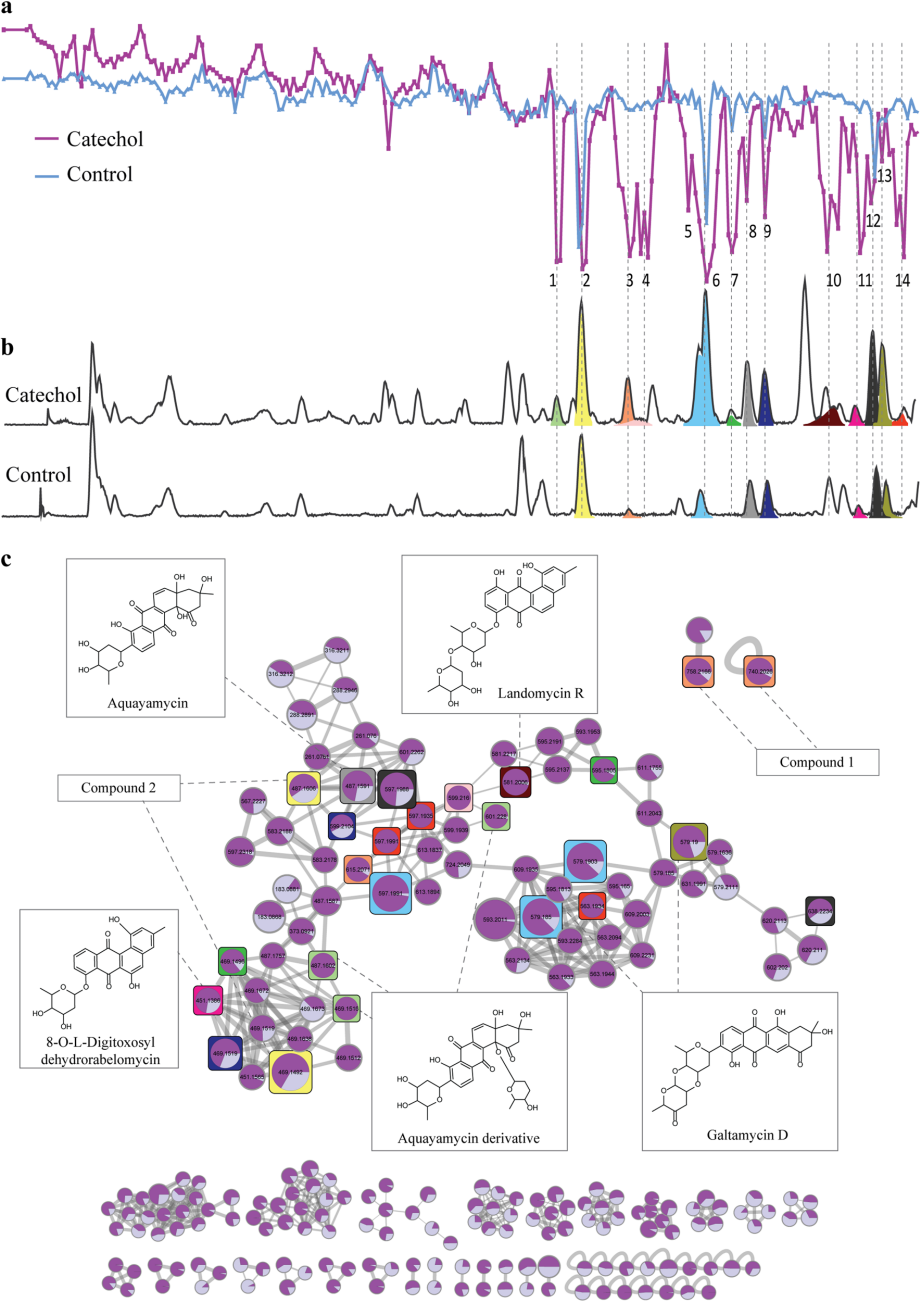
NanoRAPIDS screen of extracts of *Streptomyces* sp. MBT84 in the presence and absence of catechol. **a** Resazurin reduction assay against *B. subtilis* 168 for each nanofraction plotted against retention time. Fractions were collected with 6 s resolution onto 384-well plates after a 10 µL injection of crude extract obtained from *Streptomyces* sp. MBT84 with catechol (purple) or without (blue) at 4 mg/mL concentrations. **b** LC–MS data presented as base peak chromatogram (BPC) with extracted ion currents of the *m*/*z* values found in the MS spectra corresponding to the bioactive peaks produced in the presence (top) and absence (bottom) of catechol. **c** Molecular network representing the bioactive molecules identified by GNPS-molecular networking and MolNetEnhancer in the extracts. Colors of the squares correspond to the colors of the chromatographic peaks and highlight the mass features detected in the bioactive nanofractions. The node size represents the relative abundance of the ion in dark purple within the catechol-grown sample and in light purple in the control. The edge thickness attribute is an interpretation of the cosine similarity score, with thicker lines indicating higher similarity.

Streptomycetes typically produce a large variety of angucyclines, which are the largest family of type II polyketides, many of which with significant bioactivity as antibiotics and/or anticancer agents^20^. We, therefore, identified this molecular family as an ideal challenge for nanoRAPIDS, seeking to identify minor molecules with bioactivity without prior pre-purification. We recently showed that there is still a large chemical space that remains to be elucidated^18,32^. *Streptomyces* sp. MBT84 produces a particularly large number of angucyclines and derivatives, many of which are produced specifically when catechol is added as an elicitor to the culture media^32^. To identify the nature of the metabolites produced by *Streptomyces* sp. MBT84, in response to catechol, the strain was streaked on MM agar plates, and the metabolites were then extracted from the spent agar after 5 days of growth using ethyl acetate (EtOAc). The crude extracts of the catechol-grown cultures showed increased bioactivity against *B. subtilis* 168, as compared to those grown under control conditions^32^. To identify which mass features correlated to the increased bioactivity of *Streptomyces* sp. MBT84, crude extracts were screened with the nanoRAPIDS platform (Fig. 4).

Following nanofractionation, the chromatogram showing the bioactivity against *B. subtilis* 168 showed 14 peaks with negative maxima (Fig. 4a). Automated peak detection combined with antimicrobial activity assays allowed the selection of the individual bioactive features (Fig. 4b), which were mapped onto the enhanced feature-based molecular network. Combining the output from molecular networking, MS2LDA, and in silico annotation, the MolNetEnhancer^33^ workflow predicted an angucycline molecular family (Fig. 4c). The bioactive angucyclines were annotated by manual comparison of the molecular formula and monoisotopic mass using the Dictionary of Natural Products (Table 2). Notably, all selected features were more abundant in the extracts from cultures supplemented with catechol.

**Table 2:** List of mass features detected in the bioactive nanofractions of *Streptomyces* sp. MBT84 extract

Peak	Mass (*m/z*)	Quasi molecular ion	Molecular formula	Compound annotation	Reference
**1**	601.228	[M + H]^+^	C_31_H_37_O_12_	Aquayamycin derivative	^53^
	487.1601	[M + H-C_6_H_10_O_2_]^+^	C_25_H_27_O_10_		
	469.1515	[M + H-C_6_H_10_O_2_-H_2_O]^+^	C_25_H_25_O_9_		
**2**	487.1606	[M + H]^+^	C_25_H_27_O_10_	Aquayamycin/fridamycin A	^54^
	469.1492	[M + H-H_2_O]^+^	C_25_H_25_O_9_		
**3**	758.2166	[M + H^]+^	C_36_H_40_NO_15_S	Unknown	–
	740.2026	[M + H-H_2_O^]+^	C_36_H_38_NO_14_S		
	615.2071	[M + H]^+^	C_31_H_35_O_13_	Unknown	–
**4**	599.216	[M + H]^+^	C_31_H_35_O_12_	Saquayamycin C1/landomycin D/grincamycin K	^34,55,56^
**5**	NA				
**6**	597.199	[M + H]^+^	C_31_H_33_O_12_	Fridamycin D/saquayamycin A1/saquayamycin B1	^34,57^
	579.1902	[M + H-H_2_O]^+^	C_31_H_31_O_11_		
	579.1849	[M + H]^+^	C_31_H_31_O_11_	Galtamycin D	^32^
**7**	595.1804	[M + H]^+^	NA	Unknown angucycline-like compound	
	469.1495	[M + H]^+^	C_25_H_25_O_9_	Antibiotic PD 116740 derivative/atramycin A/landomycin I/urdamycin B derivative	^58–60^
**8**	487.159	[M + H]^+^	C_25_H_27_O_10_	Aquayamycin/fridamycin A	^36,54^
**9**	599.2103	[M + H]^+^	C_31_H_35_O_12_	Saquayamycin C1/landomycin D/grincamycin K	^34,56,61^
	469.1518	[M + H-C_6_H_10_O_3_]^+^	C_25_H_25_O_9_		
**10**	581.2005	[M + H]^+^	C_31_H_33_O_11_	Landomycin R	^62^
**11**	451.1385	[M + H]^+^	C_25_H_23_O_8_	8-O-l-Digitoxosyldehydrorabelomycin	^63^
**12**	597.1988	[M + H]^+^	C_31_H_33_O_12_	Fridamycin D/saquayamycin A1/saquayamycin B1	^34,57^
	638.2234	[M + H+MeCN]^+^	C_33_H_36_NO_12_		
**13**	579.19	[M + H]^+^	C_31_H_31_O_11_	Galtamycin D	^32^
**14**	597.1935	[M + H]^+^	C_31_H_33_O_12_	Fridamycin D/saquayamycin A1/saquayamycin B1	^34,57^
	597.199	[M + H]^+^	C_31_H_33_O_12_		
	563.1933	[M + H-H_2_O-O]^+^	C_31_H_31_O_10_		

Multiple mass features were detected in the bioactive nanofractions, mostly due to in-source fragmentation (Table 2). For example, for peak 1, three mass features were selected, namely, *m/z* 601.228, *m/z* 487.1601, and *m/z* 469.1515, that correspond to the mass of protonated aquayamycin derivative, in-source sugar loss and subsequent water loss. Two of the 14 bioactivity peaks, namely 3 and 6, were caused by co-elution of two different compounds. To our knowledge, the compounds with *m/z* 758.2166 and *m/z* 615.2071, responsible for the bioactivity of peak 3, have not been previously described. Interestingly, the mass feature with *m/z* 758.2166 did not cluster with the angucycline spectral family and was classified as an aminocyclitol glycoside based on the enhanced molecular network annotation (Fig. 4c).

Large-scale fermentation was performed to elucidate the structure of this compound. *Streptomyces* sp. MBT84 was grown confluently on 12 × 12 mm MM agar plates with 50 µM catechol, and the metabolites were extracted with EtOAc. Following chromatographic separation, **1** (red amorphous powder) and **2** (yellow amorphous powder) were isolated. Compound **1** had a molecular formula of C_36_H_39_NO_15_S, with 18 degrees of unsaturation based on high resolution-electrospray ionization-mass spectrometry (HRESIMS) analysis. Detailed NMR analysis showed that **1** is a derivative of saquayamycin B_1_ (Figs. S10–S13 in Supplementary data file 1)^34^. This derivative was named saquayamycin N. The derivatization is at C-5, which showed in the NMR a signal for a tetrasubstituted carbon (*δ*_c_ 162.6) instead of a methine group in saquayamycin B_1_. This was established through the HMBC correlations from H_2_-4 and H-6, which is now a singlet proton (*δ*_H_ 6.65, s), to C-4a and C-5 (Fig. S14 in Supplementary data file 1). An *N*-acetylcysteine moiety was identified as the remaining part of **1** based on the observed remaining NMR signals and correlations, together with the established molecular formula. The *N*-acetylcysteine was connected to C-5 of the saquayamycin B core through the sulfur atom based on the HMBC and NOESY correlations observed from the protons of the methylene group in *N*-acetylcysteine to C-5 and H-6, respectively (Figs. S14 and S15 in Supplementary data file 1). The absolute configuration of compound **1** could not be determined due to the limited amount of material purified. The configuration is most likely 3*R*, 4a*S*, 12b*S*, as was consistently observed for all saquayamycins identified so far^34,35^. For the *N*-acetylcysteine moiety, an l-cysteine would be expected. The molecular formula of compound **2** was established to be C_25_H_26_O_10_, with 13 degrees of unsaturation, based on HRESIMS analysis. 1D and 2D NMR analysis (Figs. S16–S22 in Supplementary data file 1) revealed that **2** is the previously known fridamycin A^36^.

**Fig. 5:**
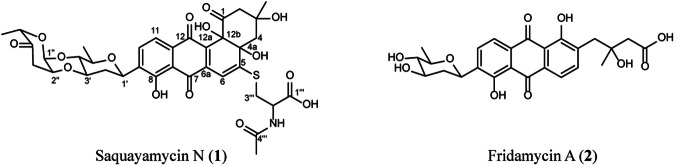
Structures of saquayamycin N (1) and fridamycin A (2) isolated from *Streptomyces* sp. MBT84. Compound **1** was named saquayamycin N. The structure of the isolated compound **2** corresponds to fridamycin A.

The structures of saquayamycin N (**1**) and fridamycin A (**2**) are shown in Fig. 5. Compounds **1** and **2** showed moderate activity against *B. subtilis* 168, with a MIC of 125 µg/mL, indicating low bioactivity. The observed bioactivity for **1** and **2** aligned with the data obtained in the resazurin reduction assay and corroborated the contribution of saquayamycin N and fridamycin A to the bioactivity observed in peaks 2 and 3, respectively (Fig. 4). The resazurin reduction assay in the nanofractionation setup only has a short incubation time, and this is radically different from—and more sensitive than—the MIC assays which are based on overnight cultures. To validate the contribution of saquayamycin N and fridamycin A to the activity detected in the crude extracts, the purified compounds were pipetted in a 384-well plate, and the resazurin reduction assay was performed as previously described. The selected concentrations were based on the MIC values, namely 125 (MIC value), 12.5, and 1.25 µg/mL concentrations (sub-MIC values). The MIC and sub-MIC concentration of 12.5 µg/mL resulted in peaks with significant negative maxima for both saquayamycin N and fridamycin A (Fig. S23).

The potential of these two isolated molecules should be addressed by further investigation of their anticancer activity, as the compounds from the angucycline family are well-known for their anticancer properties^37^.

NanoRAPIDS is a pipeline based on at-line nanofractionation technology, which enables the detection of individual mass features that contribute to the bioactivity of nanofractions. If multiple fractions with bioactivity are identified, GNPS will allow their linkage if, indeed they would belong to the same (known or unknown) family. Thus, the nanoRAPIDS platform has the potential to go beyond the discovery of known specialized metabolites and facilitates the identification of new natural product families. Nevertheless, the discovery of novel bioactive natural products remains a substantial challenge in the field of natural product discovery.

## Conclusions

The nanoRAPIDS platform is an efficient and cost-effective technology based on nanofractionation, which allows the identification of low-abundance molecules, and in particular antibiotics, in small culture extracts against a background of abundantly present known antibiotics. The powerful combination of analytical LC–MS/MS, bioassays, and open bioinformatic tools, including MZmine, GNPS Web-platform, and Cytoscape, creates an attractive pipeline for natural product discovery. Only minimal amounts of the crude extract are required for LC–MS/MS injection, allowing a significant reduction of fermentation volumes. The analysis of a single sample using the nanoRAPIDS platform requires approximately 24 h. Therefore, multiple extracts obtained from a range of culturing conditions can be rapidly tested for bioactive compounds and dereplicated, which is particularly powerful when combined with elicitation approaches in antibiotic discovery^12,13^.

Proof of concept for the technology was obtained by identification of the bioactive members of the iturin and surfactin molecular families in extracts of *Bacillus* sp. 90A-23, and then successfully applied to discover the angucycline saquayamycin N with a unique cysteine moiety in extracts of *Streptomyces* sp. MBT84. The method is versatile and can be readily applied to screen nanoscale-level extracts or biological mixtures against a range of indicator strains.

## Methods

### Bacterial strains and culturing conditions

*Bacillus* sp. 90A-23 was obtained from the Auburn strain collection and had previously been isolated from the peanut rhizosphere in Tallassee, AL, USA. The strain was fermented in 100 mL Erlenmeyer flasks with 30 mL of tryptic soy broth (TSB) at 30 °C at 220 rpm for 3 days. *Streptomyces* sp. MBT84^32^ was obtained from the Leiden University strain collection and had been isolated from soil samples collected from the Qinling mountains (Shanxi province, China^38^). *Streptomyces* sp. MBT84 was grown confluently on minimal media (MM) agar plates with and without 100 µM catechol for 5 days at 30 °C. *Escherichia coli* strain AS19-RlmA^-^^39^, *Bacillus subtilis* 168^40^, and *Aspergillus niger* N402^41^ were used as indicator strains for bioactivity assays. The bacterial strains were grown on Luria-Bertani (LB) agar at 37 °C for 24 h, *A. niger* was propagated on complete medium (CM) agar at 25 °C for 48 h.

### Metabolite extraction

Following fermentation of *Bacillus* sp. 90A-23, 1.5 g of Diaion® HP-20 (Resindion, Mitsubishi) was added to the cultures and stirred overnight at 4 °C. The resin was collected by filtration, washed with distilled water, resuspended in 30 mL methanol, and maintained at 4 °C for 4 h. The resulting methanol fraction was collected and concentrated under reduced pressure to a final concentration of 15 mg/mL. *Streptomyces* sp. MBT84 was grown on MM agar plates for 5 days, after which the agar was cut into small pieces and soaked overnight in ethyl acetate (EtOAc)^32^. The solvent was evaporated at room temperature, and the dry extracts were dissolved in MeOH to a final concentration of 4 mg/mL.

### Liquid chromatography, at-line nanofractionation and mass spectrometry

Liquid chromatography separation, subsequent at-line nanofractionation, and parallel mass spectrometry analysis were performed in an automated fashion. For LC-MS analyses, 10 μL were injected into Waters Acquity UPLC system equipped with XBridge Peptide BEH C18 column (5 µm, 300 Å, 4.6 × 100 mm). The flow rate used was 0.6 mL/min. Solvent A was 0.1% formic acid in water; solvent B was 0.1% formic acid in ACN. The gradient started with 100% A and was from the start linearly increased to 50% B over 20 min, and then linearly increased to 90% B over 5 min, then held at 90% for 5 min, and then increased to 100% B over 1 min and held at 100% B for 10 min. After the column, the flow was split at a 1:9 ratio. The smaller fraction was sent to the Waters Acquity photodiode array detector followed by a Thermo Instruments MS system (LTQ Orbitrap XL, Bremen, Germany) equipped with an electrospray ionization source (ESI). As for the MS, the following ESI parameters were used^42^: capillary voltage 5 V, spray voltage 3.5 kV, capillary temperature 300 °C, auxiliary gas flow rate 10 arbitrary units, and sheath gas flow rate 50 arbitrary units. Full MS spectra were acquired with the Orbitrap in positive mode at a mass range of 100–2000 *m/z* and FT resolution of 30,000. Data-dependent MS^2^ spectra were acquired in the ion trap for the three most intense ions using collision-induced dissociation (CID). The larger fraction was sent to the chip-based nano-electrospray ionization source/fractionation robot (NanoMate Triversa, Advion BioSciences). The nanofractions were collected every six seconds onto black 384-well plates (Greiner Bio One, Alphen aan den Rijn, The Netherlands) in a serpentine pattern. Each sample was collected into 350 wells of the 384-well plate. After the nanofractionation, the plates were dried under vacuum. In order to determine the delay between the MS trace and the bioassay trace, nalidixic acid was dissolved in distilled water to a final concentration of 0.5 mg/mL, and 1 μL was fractionated (Fig. S1).

### Resazurin reduction assay

The antibacterial and antifungal activities of the nanofractionated *Bacillus* sp. 90A-23 extract was determined in a resazurin reduction assay. Indicator strains *B. subtilis* 168 and *E. coli* ASD19 were cultured in liquid LB medium at 37 °C to exponential phase and diluted in LB to the final OD_600_ of 0.005. Resazurin solution was then added to the cells to a final concentration of 15 μg/mL, and 25 μL of this mixture was directly pipetted into each well of 384-well plates containing dried nanofractions. Controls were pipetted in the last column of the plate: positive control (cells with resazurin and ampicillin (0.2 mg/mL)), growth control (cells and resazurin), blank (LB with resazurin), and sterility control (only LB). Directly after this, the plates were spun down for 10 s at 1500 rpm and subsequently incubated without the lids in the plastic bags in a humidified incubator at 37 °C for 2–3 h^21^.

Spores of *A. niger* N402 were harvested from CM agar plates, pregerminated in complete medium for 5 h at 30 °C, and diluted in CM to the final titer of 1 × 10^5^ spores/mL. Each well of 384-well plate with dried nanofraction was filled with 25 µL of *A. niger* spore solution. Amphotericin B (0.02 mg/mL) was added to the *A. niger* spores in the positive control wells. Subsequently, the plates were centrifuged and incubated at 30 °C overnight, as explained above. Finally, resazurin was added to each well to a final concentration of 60 μg/mL and incubated for 45 min at 37 °C. After the incubation, the fluorescence was measured as a single point measurement using a Victor3 Plate Reader (PerkinElmer, Inc., Waltham, MA, USA) at 550 nm excitation and 590 nm emission wavelengths. The fluorescent readout was normalized by dividing each value measured with the median of all the values obtained in a single measurement. Subsequently, the bioactivity chromatograms were plotted in GraphPad Prism 8 software (La Jolla, CA, USA) in a graph showing the normalized response of each nanofraction versus the time at which each nanofraction was collected.

### Alignment of chromatograms

Alignment of the different chromatographic plots was conducted through a semi-automated process. Initially, the delay introduced by the tubing setup was determined by the injection of 0.5 μg of nalidixic acid in the nanoRAPIDS set-up, and *E. coli* ASD 19 was used as the indicator strain (Figure S1). Once the delay was quantified in 0.3 min, and the bioactivity chromatogram was adjusted based on this determined delay. Subsequently, both the mass spectrometry (MS) chromatogram, utilizing automatic peak detection, and the bioactivity chromatogram were standardized to the same size. Given the identical time function, the chromatograms were then aligned to the center, ensuring accurate synchronization of the two datasets. This semi-automated alignment procedure aimed to enhance precision in data interpretation and visualization, facilitating meaningful comparisons between the MS and bioactivity chromatograms.

### Feature-based GNPS networking

A molecular network was created with the feature-based molecular networking workflow^22^ using Global Natural Product Social molecular networking (GNPS)^15^. Prior to feature-based GNPS networking, the MS files in mzXML format were imported into MZmine 2.53^23^ for data processing. Mass ion peaks were detected using the centroid algorithm with a noise level set to 1.0 × 10^4^ for MS scans and 0 for MS^2^ scans. Afterwards, chromatograms were built for the detected masses with a minimum group size of 10, *m/z* tolerance of 0.001 *m/z*, group intensity threshold of 1.0 × 10^4^, and a minimum height of 5.0 × 10^4^. Chromatogram deconvolution was then performed using a local minimum search algorithm (search minimum in RT range 0.1 min, chromatographic threshold 90%, minimum relative height 1%, minimum absolute height 1.0 × 10^4^, minimum ratio of peak top/edge two and peak duration range 0.05–3 min). A feature with its MS^2^ scans was paired using a 0.05 *m/z* range and retention time range of 1 min. In the generated peak lists, isotopes were identified using isotopic peaks grouper (*m*/*z* tolerance 0.001 *m/z* and retention time tolerance 0.1 min). Duplicate peaks were filtered using the single feature and old average mode by deleting the duplicate rows with *m/z* tolerance of 0.001 *m/z* in an RT range of 0.05 min and with an *m/z* tolerance of 1 *m/z* in an RT range of 0.05 min, respectively. The resulting peak list was filtered with a rows filter to keep only the peaks with MS^2^ scans. The filtered peak list was exported to the feature quantification table and the MS^2^ spectral summary file for feature-based molecular networking. On GNPS, the precursor ion mass tolerance was set to 0.02 Da and the MS/MS fragment ion tolerance to 0.9 Da. A molecular network was then created where edges were filtered to have a cosine score above 0.7 and more than three matched peaks. All matches kept between network spectra and library spectra were required to have a score above 0.7 and at least 3 matched peaks. To enhance chemical structural information within the molecular network, information from in silico structure annotations from GNPS Library Search, Network Annotation Propogation^43^, Dereplicator^44^ were incorporated into the network using the GNPS MolNetEnhancer workflow^33^. Chemical class annotations were performed using the ClassyFire chemical ontology^45^. The molecular networks were visualized using Cytoscape^24^ software. The molecular networking job from the extract of *Bacillus* sp. 90A-23 can be publicly accessed at https://gnps.ucsd.edu/ProteoSAFe/status.jsp?task=1ba65a809b17413694522c27b0d952df. The molecular networking job from the extract of *Streptomyces* sp. MBT84 can be publicly accessed at https://gnps.ucsd.edu/ProteoSAFe/status.jsp?task=15e986d4356f41b4962baab398c571fa. LC–MS/MS data were deposited in the MassIVE Public GNPS data set (MSV000094019).

### Large-scale fermentation, extraction, and fractionation

Large-scale fermentation of *Streptomyces* sp. MBT84, extraction, and fractionation were performed as previously described^32^. Briefly, *Streptomyces* sp. MBT84 was grown on solid MM supplemented with 50 µM catechol, 1% glycerol, and 0.5% mannitol at 30 °C for five days. Agar plates were extracted with EtOAc, and 1.6 g of crude extract was obtained. This extract was adsorbed onto 1.6 g silica gel (pore size 60 Å, 70–230 mesh, Sigma Aldrich), and loaded on a silica column, followed by gradient elution using mixtures of n-hexane, EtOAc, and MeOH. The fractions that eluted with 50% MeOH: 50% EtOAc were combined with the fraction that eluted with 100% MeOH and reconstituted in 50% ACN: 50% H_2_O. The resulting fraction was subjected to a SunFire C_18_ column (10 μm, 100 Å, 19 × 150 mm) and eluted with H_2_O (solvent A) and ACN (solvent B) with 0.1% formic acid in a gradient of solvent B 30–70% in 30 min, at a flow rate of 15 mL/min. Fractions A–C were collected every 10 min. The fraction A was further purified on a semi-preparative SunFire C_18_ column (5 μm, 100 Å, 10 × 250 mm), run at 3 mL/min, and eluted using a H_2_O and ACN with 0.1% formic acid gradient of 47–50% in 20 min, to yield compound **1** (0.8 mg) and compound **2** (2.6 mg). NMR data were acquired on Bruker Ascend 850 NMR spectrometer (850 MHz) and interpreted using the MestreNova V.14 software.

Saquayamycin N (**1**): red amorphous powder; ^1^H and ^13^C NMR data, see Table S1; HRESIMS (positive mode) *m/z* 758.2103 [M  +  H]^+^ (calcd. for C_36_H_40_NO_15_S, 758.21187).

Fridamycin A (**2**): yellow amorphous powder; ^1^H and ^13^C NMR data, see Table S2; HRESIMS (positive mode) *m/z* 487.1606 [M  +  H]^+^ (calcd. for C_25_H_27_O_10_, 487.1604).

### MIC tests

The minimum inhibitory concentration (MIC) was determined by the broth microdilution method using the British Standard BS EN ISO 20776–1:2006^46–49^. A stock solution of compounds **1** and **2** was made by dissolving them in MeOH to a concentration of 1 mg/mL. *B. subtilis* 168 was grown from an overnight culture until an OD_600_ of 0.3 in LB and diluted until a concentration of bacteria of 1 × 10^6^ CFU/mL in fresh broth. Two-fold serial dilutions of compounds **1** and **2** were tested in the concentration range of 250–0.12 μg/mL in triplicates. Growth control wells containing 100 μL of 5 × 10^5^ CFU/mL were included without test compounds. After overnight incubation at 37 °C, inhibition was defined as no visible growth compared to the growth observed in the control wells.

### Reporting summary

Further information on research design is available in the Nature Portfolio Reporting Summary linked to this article.

### Supplementary information

Supplementary Information

Description of Additional Supplementary File

Supplementary Data 1

Reporting Summary

## Data Availability

All datasets generated and/or analyzed during the current study are publicly available. The metabolomics dataset is available in the GNPS repository. The data from the extract of *Bacillus* sp. 90A-23 can be publicly accessed at https://gnps.ucsd.edu/ProteoSAFe/status.jsp?task=1ba65a809b17413694522c27b0d952df. The molecular networking job from the extract of *Streptomyces* sp. MBT84 can be accessed at https://gnps.ucsd.edu/ProteoSAFe/status.jsp?task=15e986d4356f41b4962baab398c571fa. LC–MS/MS data and metadata were deposited in the MassIVE Public GNPS data set (MSV000094019). Copies of the NMR spectra of the metabolites identified in this study are provided in the Supplementary Data 1 file, and the raw data are available upon request.
