# Supplementary material for: nanoRAPIDS as an analytical pipeline for the discovery of novel bioactive metabolites in complex culture extracts at the nanoscale
Source: Commun Chem. 2024 Apr 1;7:71. doi: 10.1038/s42004-024-01153-y (PMC10984978; doi:10.1038/s42004-024-01153-y)
Supplement: Supplementary file 1 — Supplementary Information [file 42004_2024_1153_MOESM1_ESM.pdf]

SUPPLEMENTAL INFORMATION

nanoRAPIDS as an analytical pipeline for the discovery of novel bioactive metabolites in complex culture extracts at the nanoscale

Isabel Nuñez Santiago[a,*], Nataliia V. Machushynets [a,*], Marija Mladic[a,b], Doris A. van Bergeijk[c,d], Somayah S. Elsayed[a], Thomas Hankemeier[e], Gilles P. van Wezel[a,#]

[a] *Molecular Biotechnology, Institute of Biology, Leiden University, Leiden,The Netherlands*

[b] *DSM-Firmenich, Delft, The Netherlands*

[c] *KU Leuven, Department of Microbiology, Immunology and Transplantation, Laboratory of Molecular Bacteriology, Leuven, Belgium*

[d] *VIB, Center for Microbiology, Leuven, Belgium*

[e] *Leiden Academic Centre for Drug Research (LACDR), Leiden University, Leiden, The Netherlands.*

* These authors contributed equally.

[#] Author for correspondence. Tel: +31 71 5274310; email: g.wezel@biology.leidenuniv.nl

Table S1. NMR data of **1** (in CD_3OD at 298 K)

Position	δ_H, mult. (J in Hz)^a	δ_C, type^b
**1**		206.7, C
**2**	a: 2.83, d (13.2)<br>b: 2.64, dd (13.2, 2.5)	53.3, CH_2
**3**		77.3, C
**4**	2.11, m	46.3, CH_2
**4a**		84.4, C
**5**		162.6, C
**6**	6.65, s	107.4, CH
**6a**		139.6, C
**7**		190.0, C
**7a**		115.5, C
**8**		158.7, C
**9**		138.2, C
**10**	7.86, d (7.8)	134.5, CH
**11**	7.55, d (7.8)	119.7, CH
**11a**		132.6, C
**12**		183.3, C
**12a**		135.3, C
**12b**		79.5, C
**3-Me**	1.24, d (1.2)	30.2, CH_3
**1′**	5.01, d (11.1)	72.6, CH
**2′**	a: 2.37, m<br>b: 1.51, m	37.8, CH_2
**3′**	3.84, m	77.9, CH
**4′**	3.49, t (9.0)	75.7, CH
**5′**	3.57, m	75.6, CH
**6′**	1.35, d (6.0)	17.7, CH_3
**1″**	5.20, d (2.8)	92.8, CH
**2″**	4.37, m	72.6, CH
**3″**	a: 2.79, dd (17.4, 2.7)<br>b: 2.55, dd (17.4, 3.7)	40.9, CH_2
**4″**		210.1, C
**5″**	4.72, q (6.8)	78.9, CH
**6″**	1.31, d (6.8)	16.5, CH_3
**1‴**		173.4, C
**2‴**	4.81, m	51.9, CH
**3‴**	a: 3.59, m<br>b: 3.21, m	33.5, CH_2
**4‴**		173.4, C
**5‴**	2.03, s	22.5, CH_3

^{a} ^{1}H 850 MHz, ^{b} ^{13}C 213 MHz

Table S2. NMR data of **2** (in CD_3OD at 298 K)

**Position**	**δ_H, mult. (*J* in Hz)^a**	**δ_C, type^b**
**1**		175.4, C
**2**	2.54, d (5.7)	46.4, CH_2
**3**		73.0, C
**4**	a: 3.10, d (13.6)<br>b: 3.01, d (13.6)	41.2, CH_2
**4a**		136.1, C
**5**	7.70, m	140.8, CH
**6**	7.68, m	119.6, CH
**6a**		132.9, C
**7**		189.2, C
**7a**		116.5, C
**8**		159.7, C
**9**		139.6, C
**10**	7.83, d (7.8)	134.2, CH
**11**	7.72, d (7.8)	120.1, CH
**11a**		133.1, C
**12**		189.1, C
**12a**		116.6, C
**12b**		162.4, C
**3-Me**	1.30, s	27.0, CH_3
**1′**	4.85^c	72.5, CH
**2′**	a: 2.45, ddd (12.9, 4.9, 2.1)<br>b: 1.41, m	40.9, CH_2
**3′**	3.71, ddd (11.3, 8.9, 4.9)	73.6, CH
**4′**	3.06, t (8.9)	78.8, CH
**5′**	3.46, dq (8.9, 6.0)	77.7, CH
**6′**	1.40, d (6.0)	18.7, CH_3

^{a} ^{1}H 850 MHz, ^{b} ^{13}C 213 MHz, ^{c} the signal was suppressed with the water peak suppression

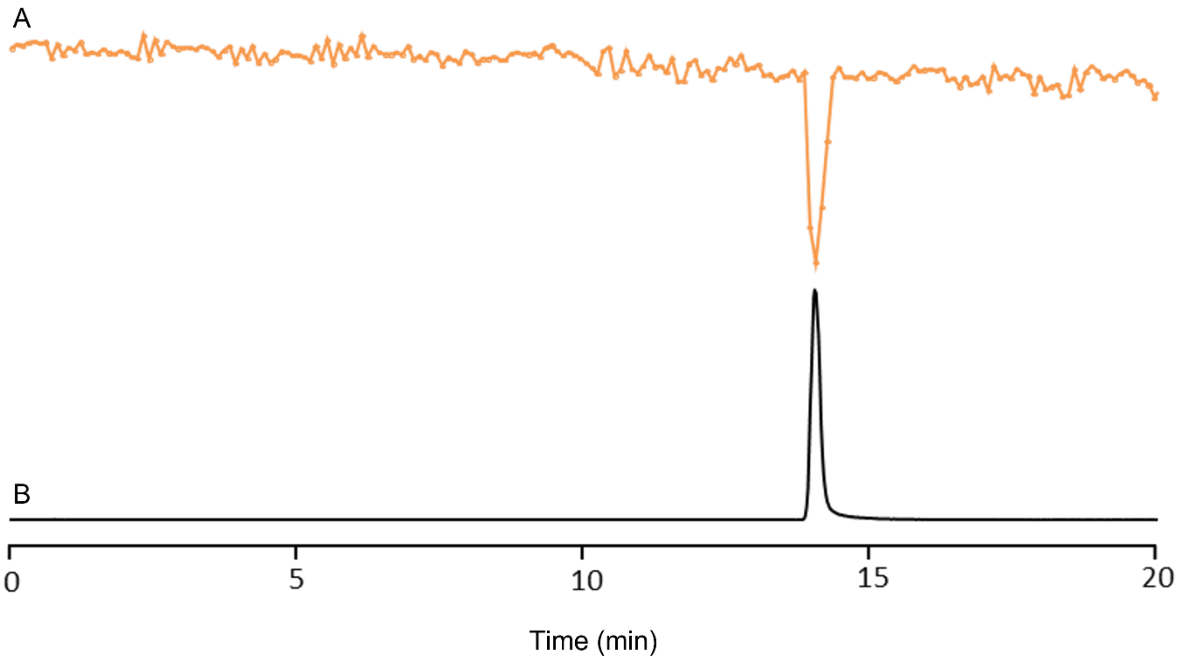

Figure S1. Delay calculation by correlation of mass spectrometry data with bioassay data. **A**. The bioactivity chromatogram of fractionated nalidixic acid against *E. coli* ASD19. The peak with the negative maxima indicates the growth inhibition of *E. coli*. **B.** The extracted ion chromatogram (EIC) of nalidixic acid. The time difference between the peak of bioactivity and MS chromatogram is determined as 0.3 min.

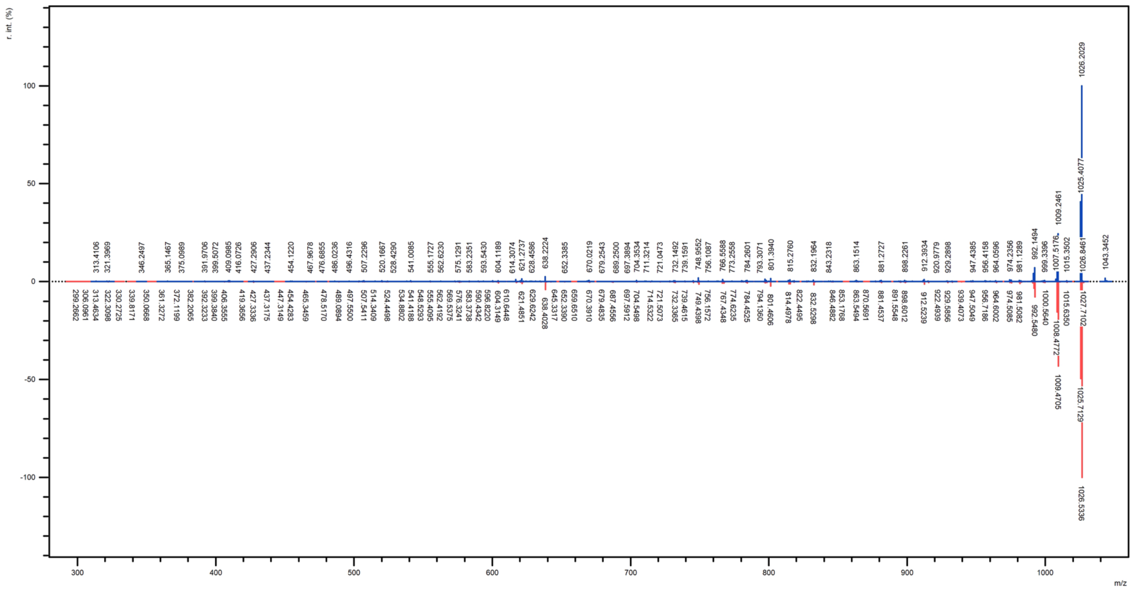

Figure S2. Direct MS/MS comparison of iturin A2 from the extract of *Bacillus* sp. 90A-23 with iturin A2 spectrum from GNPS spectral library (CCMSLIB00000086198).

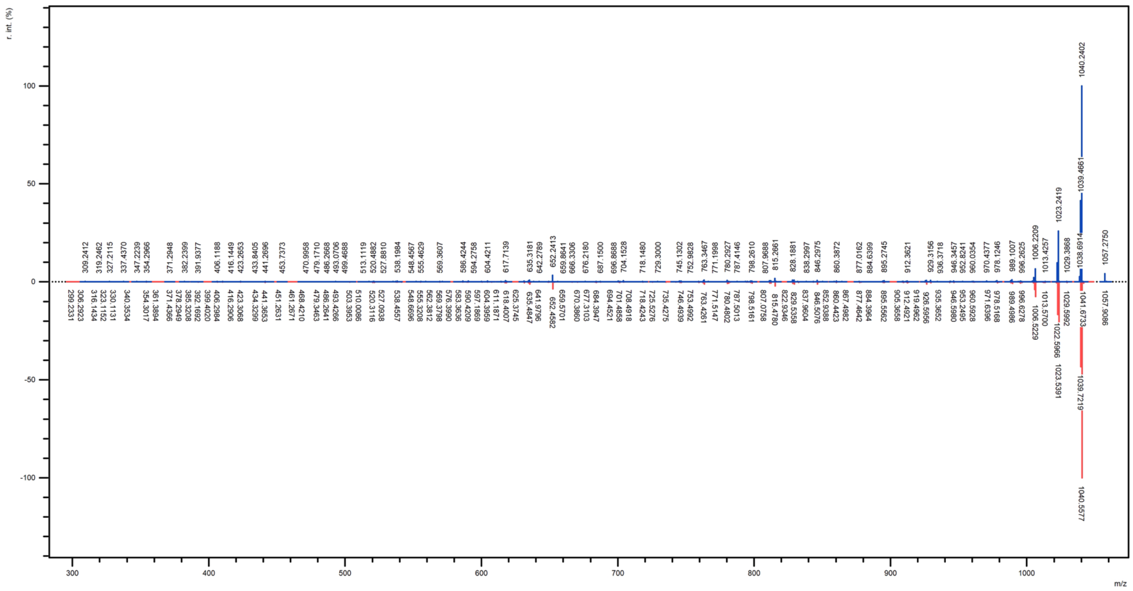

Figure S3. Direct MS/MS comparison of iturin A3/A4/A5 from the extract of *Bacillus* sp. 90A-23 with iturin A3/A4/A5 spectrum from GNPS spectral library (CCMSLIB00000086200).

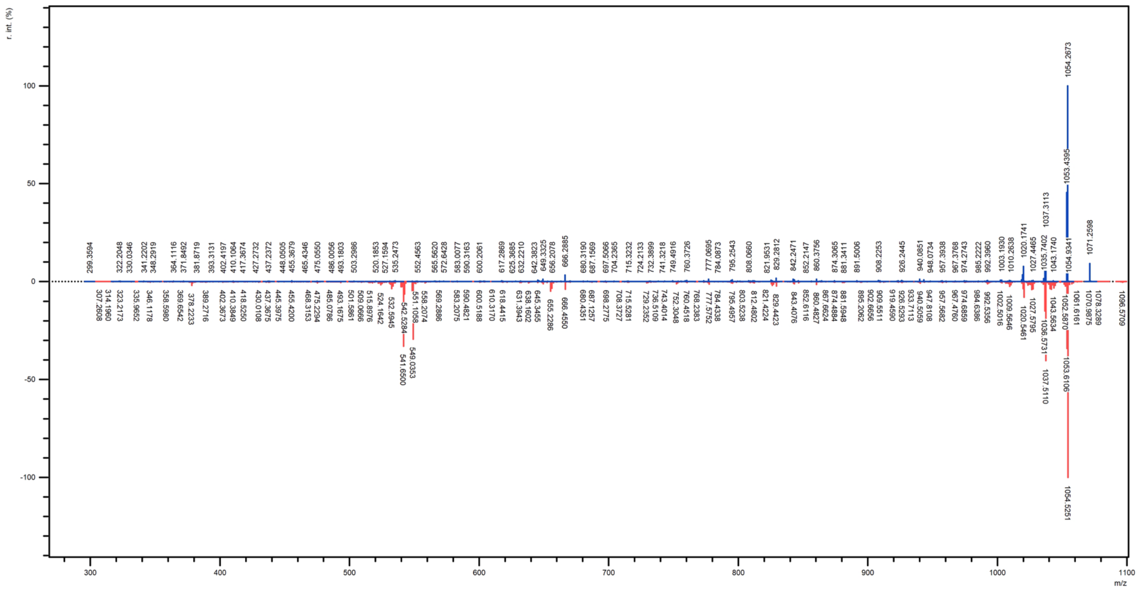

Figure S4. Direct MS/MS comparison of iturin A6/7 from the extract of *Bacillus* sp. 90A-23 with iturin A6/7 spectrum from GNPS spectral library (CCMSLIB00000086202).

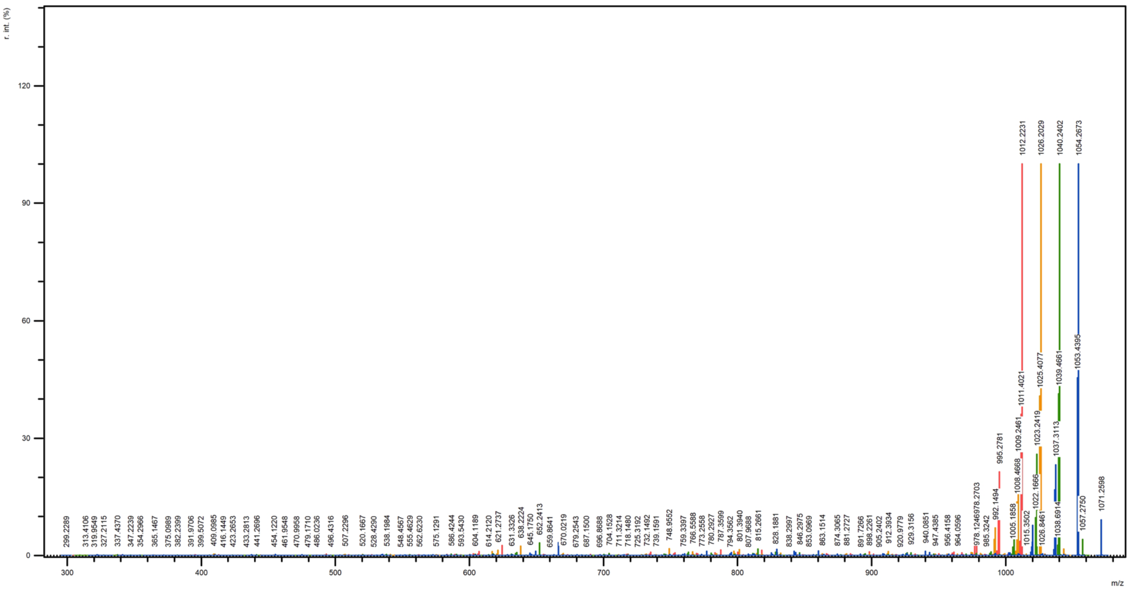

Figure S5. Direct MS/MS comparison of the iturin A1, iturin A2, iturin A3/A4/A5 and iturin A6/7 spectra from *Bacillus* sp. 90A-23 extract.

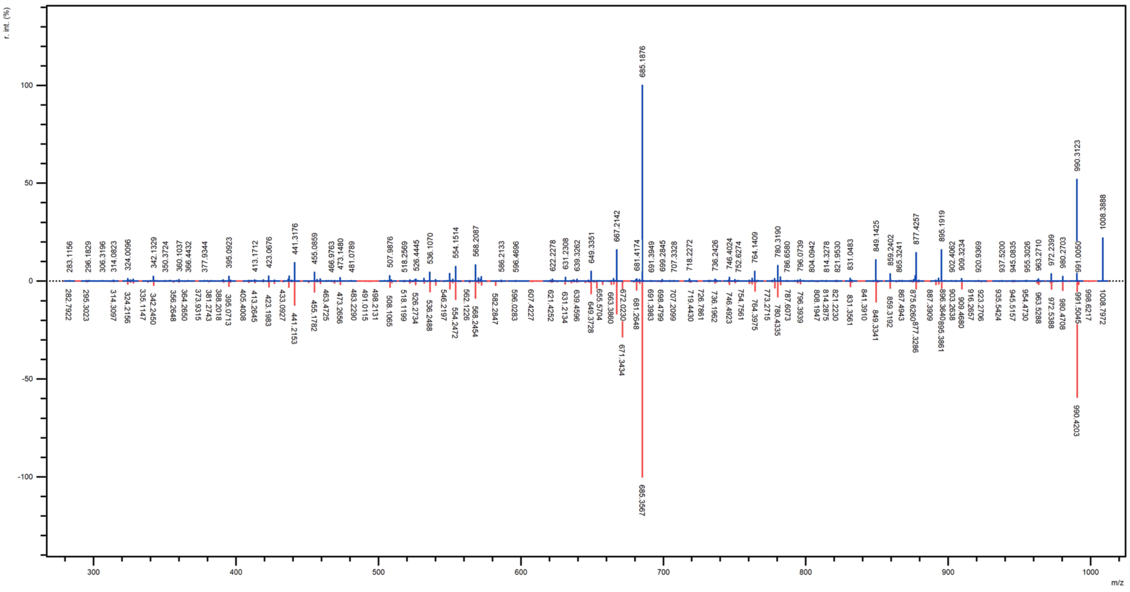

Figure S6. Direct MS/MS comparison of surfactin Leu/Ile[7] C13 from the extract of *Bacillus* sp. 90A-23 with surfactin Leu/Ile[7] C13 spectrum from GNPS spectral library (CCMSLIB00000006885).

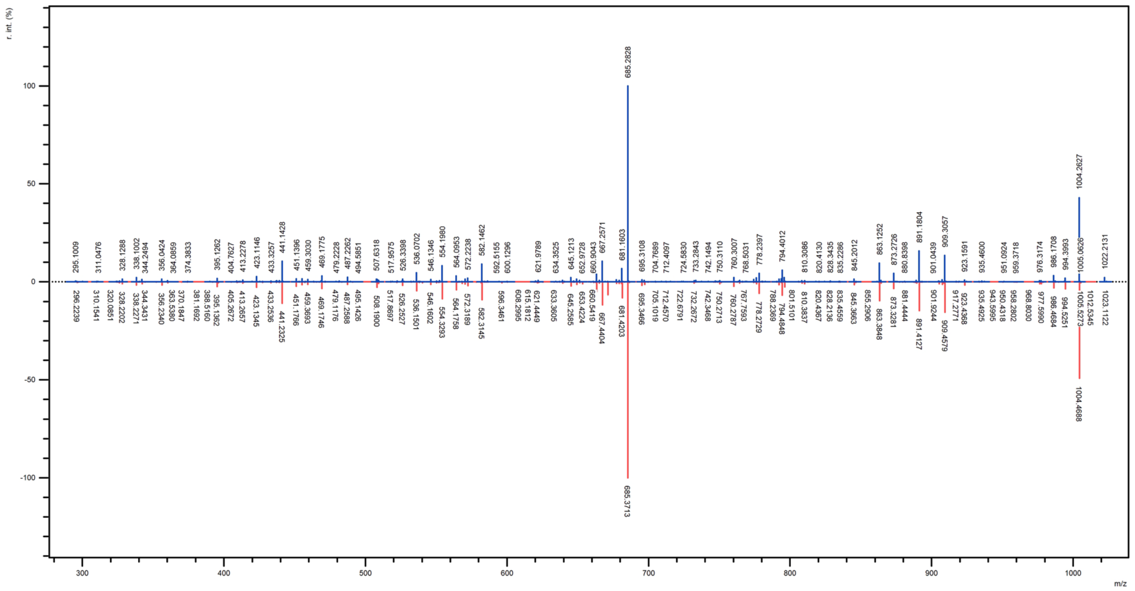

Figure S7. Direct MS/MS comparison of surfactin Leu/Ile^{7} C14 from the extract of *Bacillus* sp. 90A-23 with surfactin Leu/Ile^{7} C14 spectrum from GNPS spectral library (CCMSLIB00000006890).

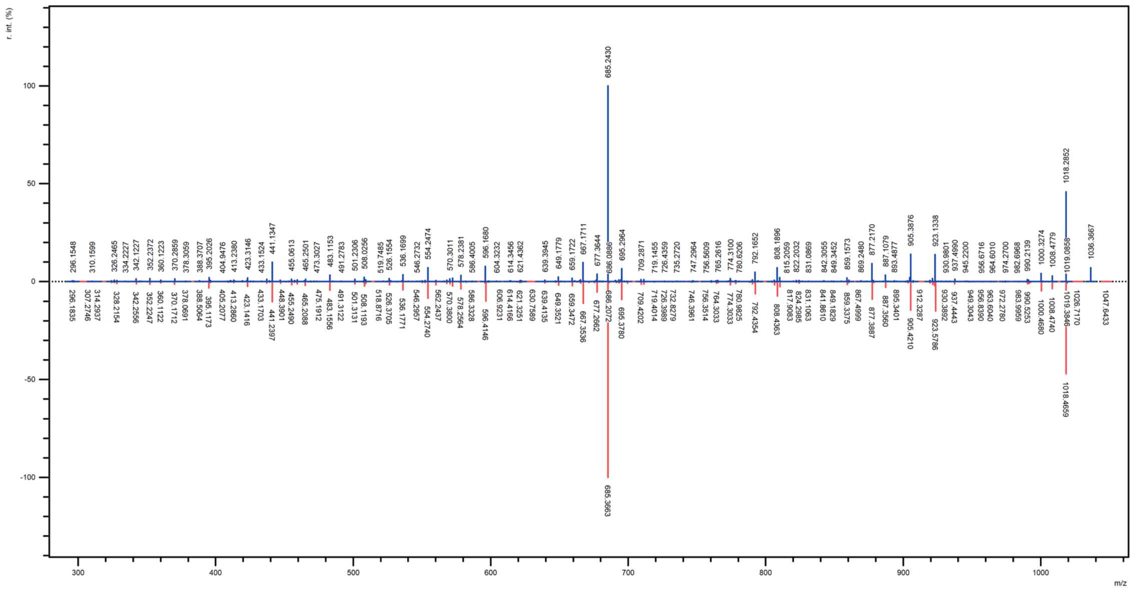

Figure S8. Direct MS/MS comparison of surfactin Leu/Ile^{7} C15 from the extract of *Bacillus* sp. 90A-23 with surfactin Leu/Ile^{7} C15 spectrum from GNPS spectral library (CCMSLIB00000006895).

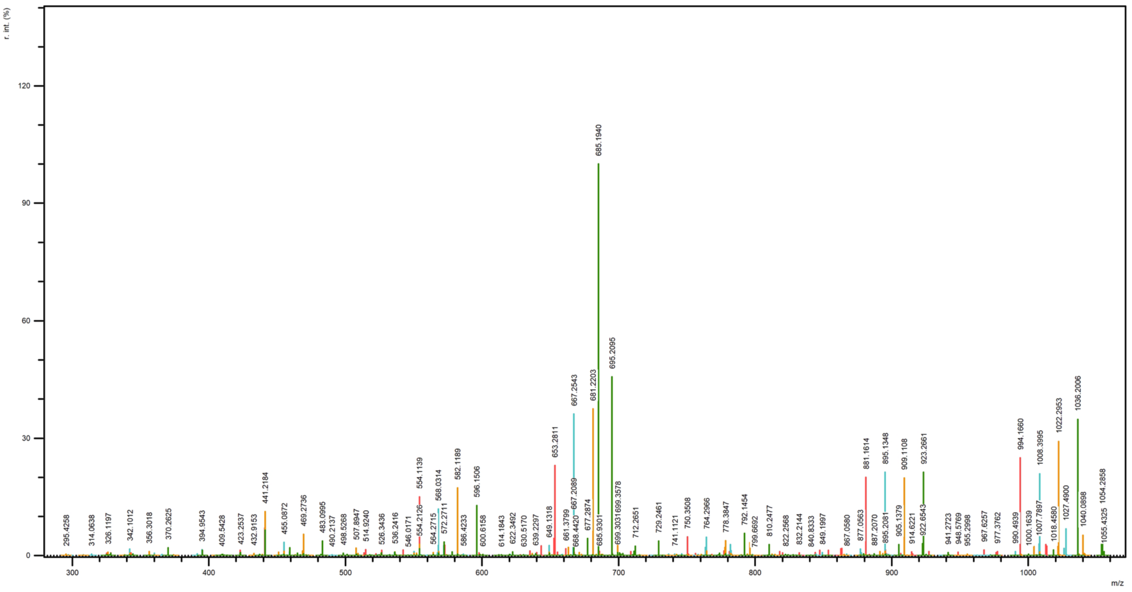

Figure S9. Direct MS/MS comparison of the C12, C13, C14 and C15 linear surfactins Leu/Ile[7] from *Bacillus* sp. 90A-23 extract.

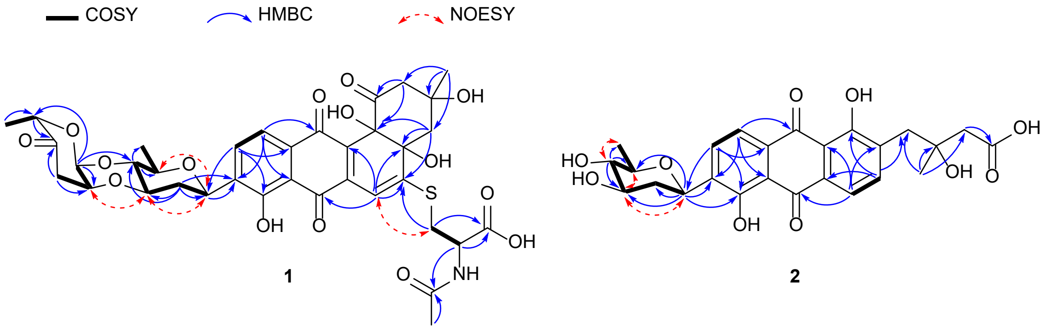

Figure S22. Correlations obtained by COSY, HMBC and NOESY measurements in NMR of saquayamycin N (**1**) and fridamaycin A (**2**).

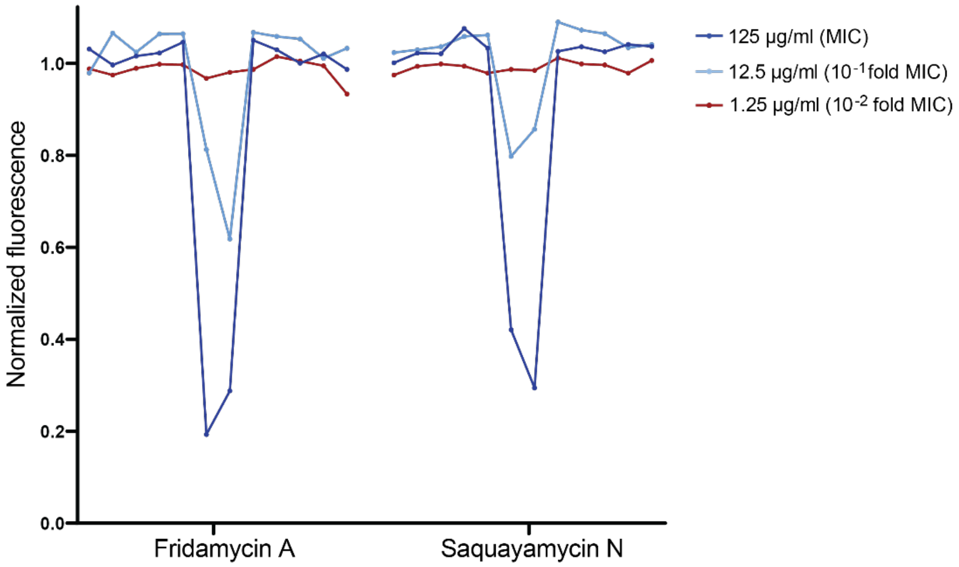

Figure S23. Results of the resazurin reduction assay for the purified saquayamycin N (**1**) and fridamycin A (**2**) against *B. subtilis* 168. Compounds were tested in 125 µg/ml, 12.5 µg/ml and 1.25 µg/ml concentration. Note, that the MIC and sub-MIC concentration (12.5 µg/ml) resulted in the drop of fluorescence.
