# Supplementary material for: nanoRAPIDS as an analytical pipeline for the discovery of novel bioactive metabolites in complex culture extracts at the nanoscale
Source: Commun Chem. 2024 Apr 1;7:71. doi: 10.1038/s42004-024-01153-y (PMC10984978; doi:10.1038/s42004-024-01153-y)
Supplement: Supplementary file 2 — Description of Additional Supplementary File [file 42004_2024_1153_MOESM2_ESM.pdf]

Description of Additional Supplementary File

File name: Supplementary Data 1

Description: NMR spectra of the metabolites identified in this study
