# Supplementary material for: nanoRAPIDS as an analytical pipeline for the discovery of novel bioactive metabolites in complex culture extracts at the nanoscale
Source: Commun Chem. 2024 Apr 1;7:71. doi: 10.1038/s42004-024-01153-y (PMC10984978; doi:10.1038/s42004-024-01153-y)
Supplement: Supplementary file 3 — Supplementary Data 1 [file 42004_2024_1153_MOESM3_ESM.pdf]

## Supplementary Data 1

**nanoRAPIDS as an analytical pipeline for the discovery of novel bioactive metabolites in complex culture extracts at the nanoscale**


Isabel Nuñez Santiago[a,*], Nataliia V. Machushynets [a,*], Marija Mladic[a,b], Doris A. van Bergeijk[c,d], Somayah S. Elsayed[a], Thomas Hankemeier[e], Gilles P. van Wezel[a,#]

[a] *Molecular Biotechnology, Institute of Biology, Leiden University, Leiden,The Netherlands*

[b] *DSM-Firmenich, Delft, The Netherlands*

[c] *KU Leuven, Department of Microbiology, Immunology and Transplantation, Laboratory of Molecular Bacteriology, Leuven, Belgium*

[d] *VIB, Center for Microbiology, Leuven, Belgium*

[e] *Leiden Academic Centre for Drug Research (LACDR), Leiden University, Leiden, The Netherlands.*

* These authors contributed equally.

[#] Author for correspondence. Tel: +31 71 5274310; email: g.wezel@biology.leidenuniv.nl

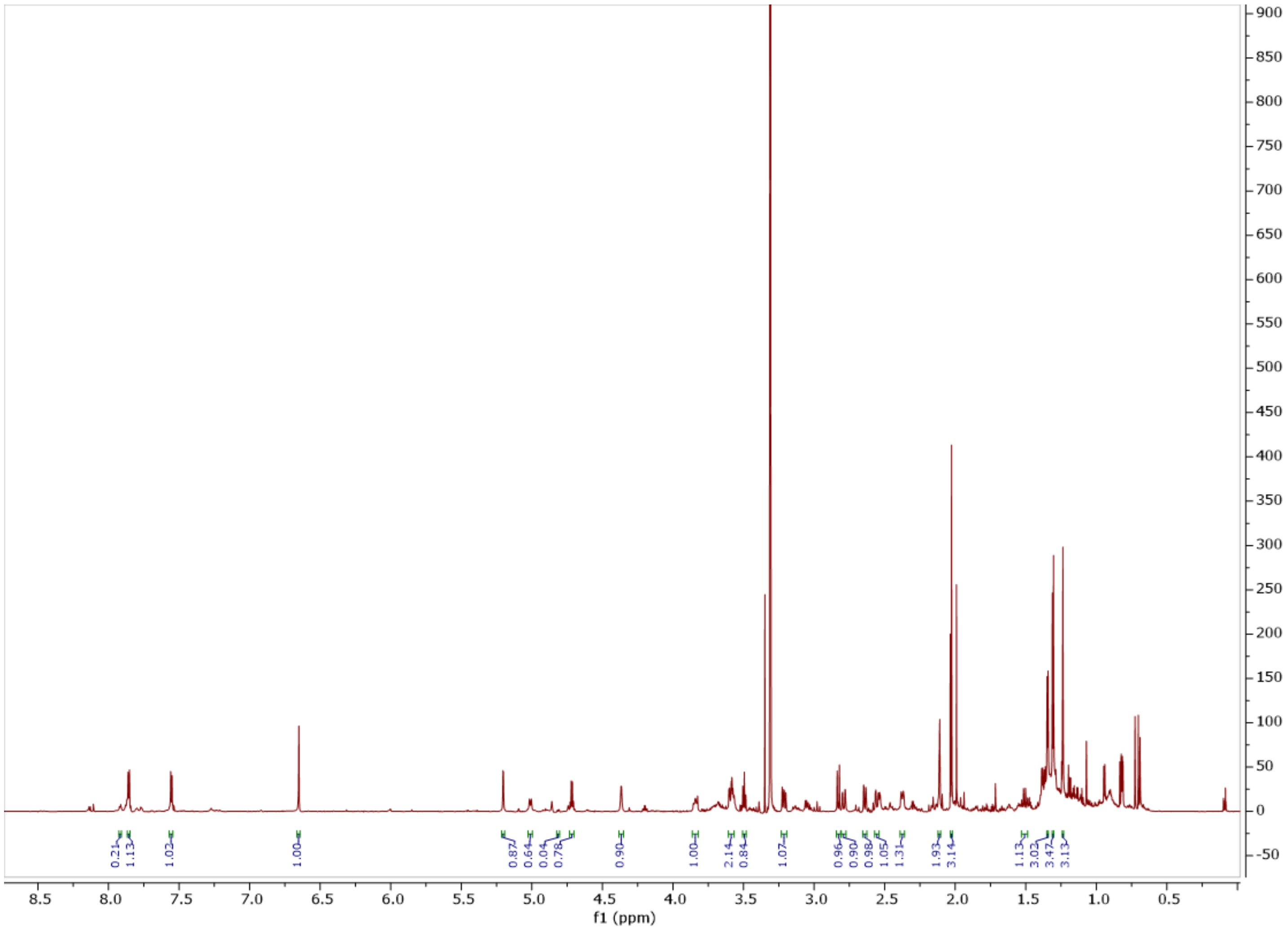


**Figure S10.** $^{1}$H NMR spectrum of saquayamycin N (**1**) (850 MHz, in $CD_3OD$).

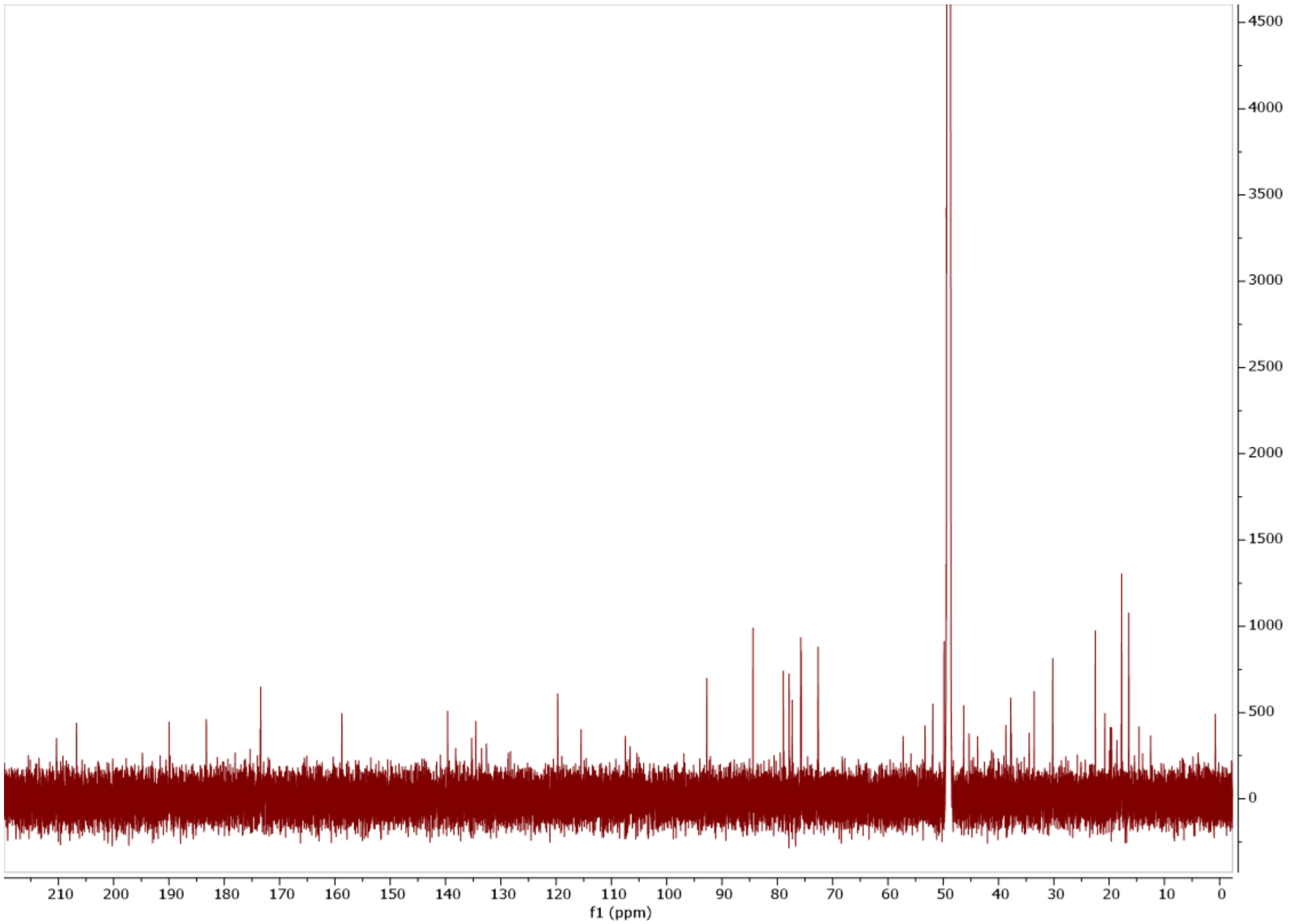


**Figure S11.** $^{13}C$ NMR spectrum of saquayamycin N (**1**) (213 MHz, in $CD_3OD$).

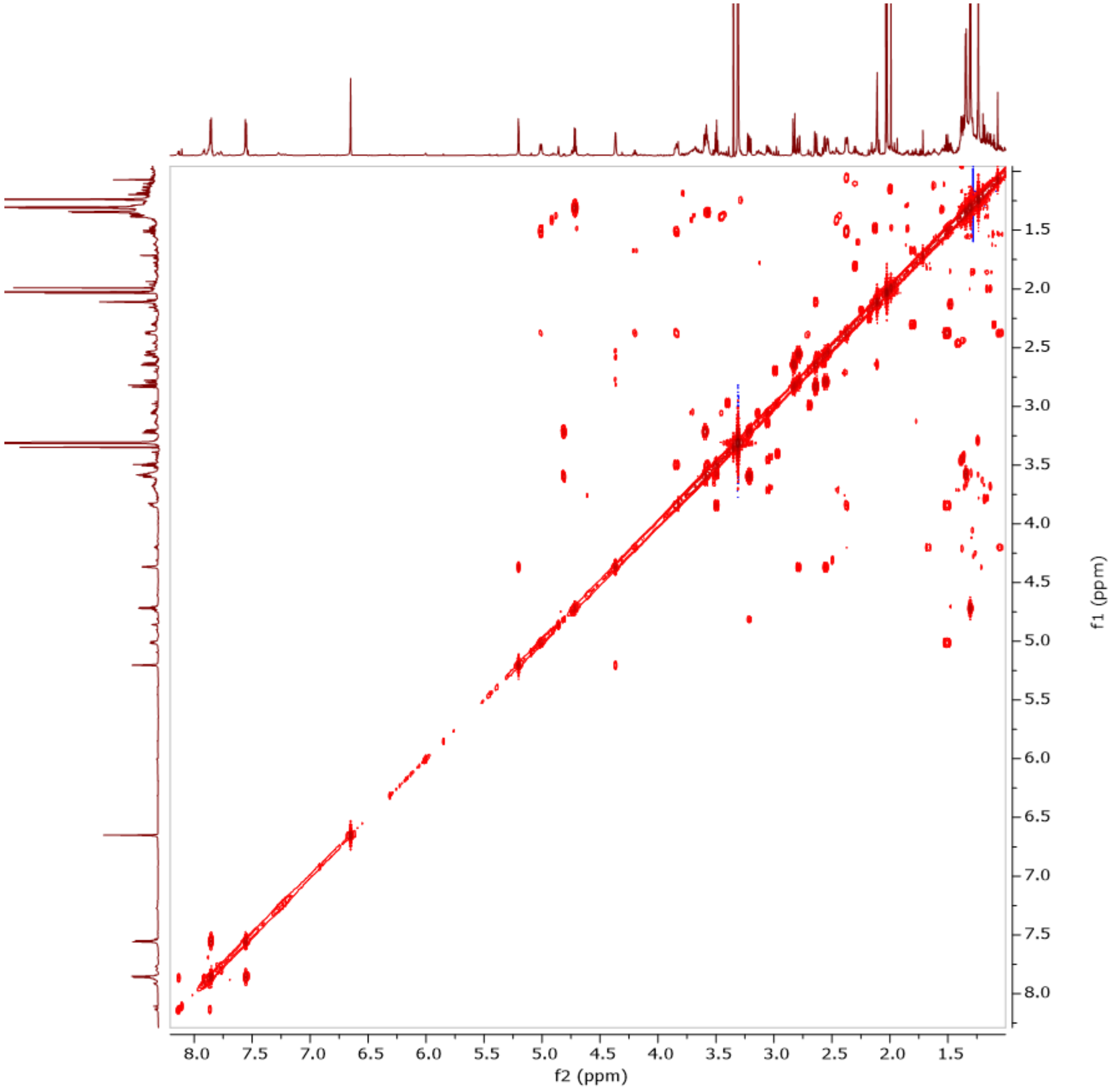


**Figure S12.** COSY spectrum of saquayamycin N (**1**) (850 MHz, in $CD_3OD$).

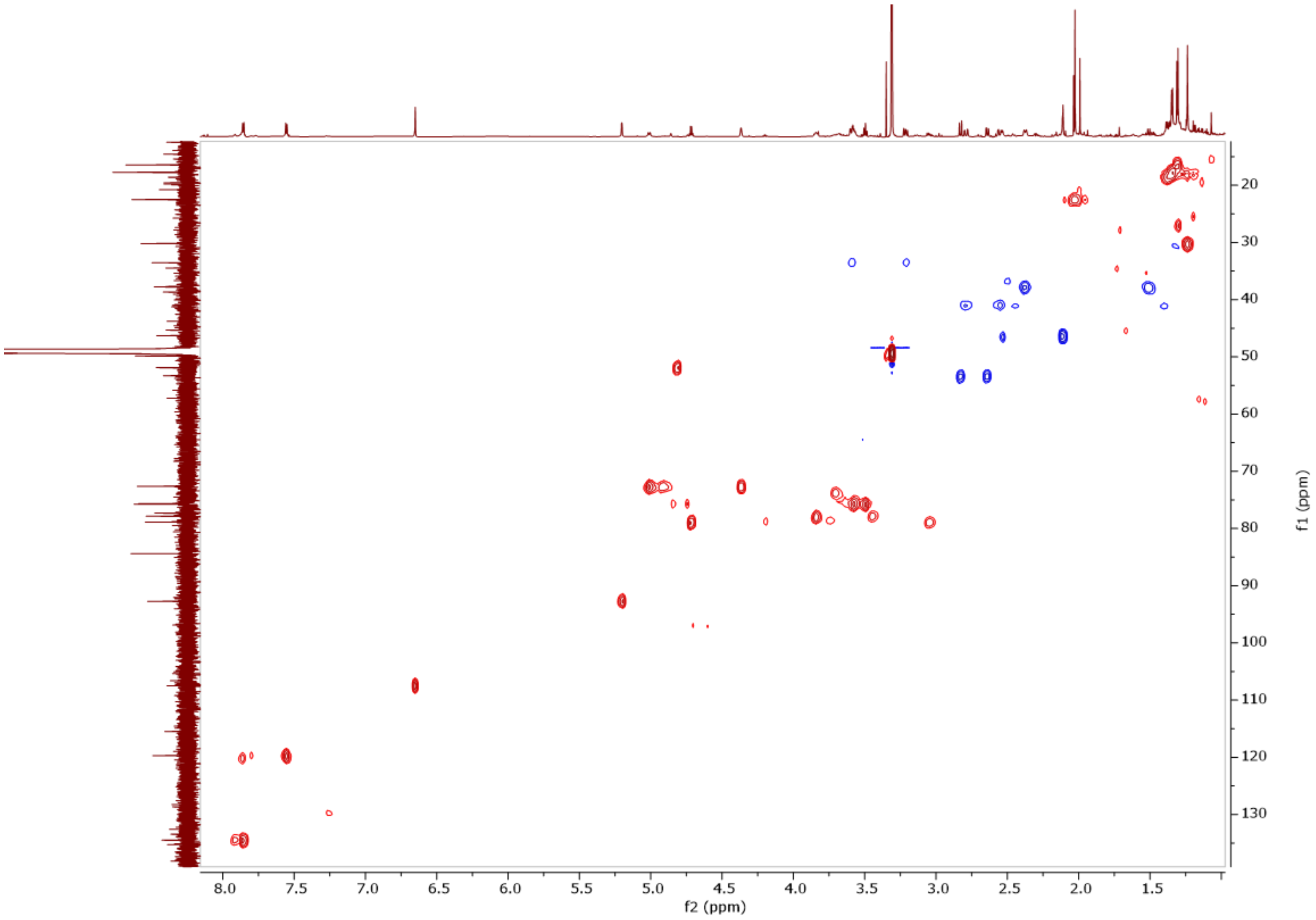


**Figure S13.** Multiplicity-edited HSQC spectrum of saquayamycin N (**1**) (850 MHz, in $CD_3OD$).

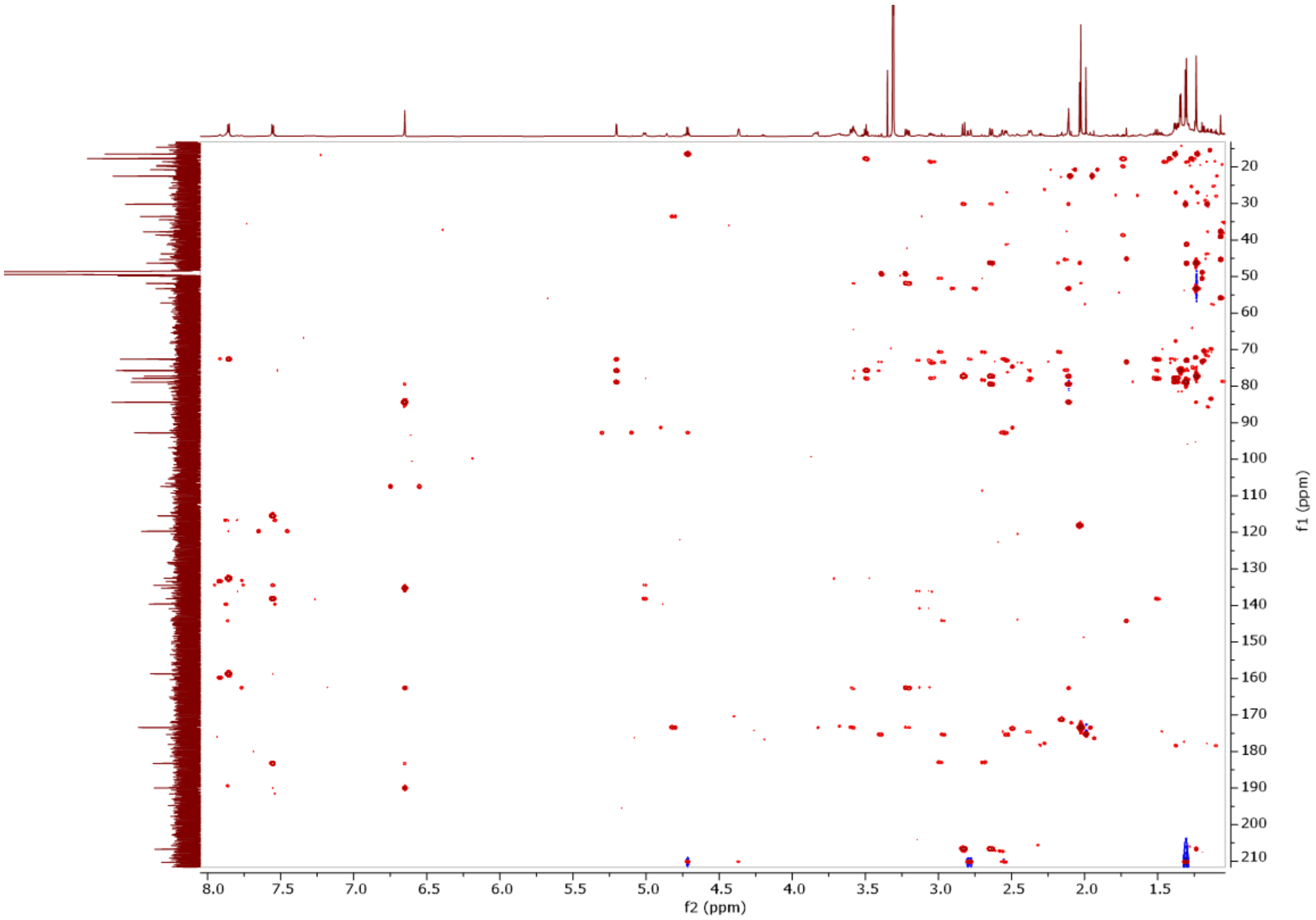


**Figure S14.** HMBC spectrum of saquayamycin N (**1**) (850 MHz, in $CD_3OD$).

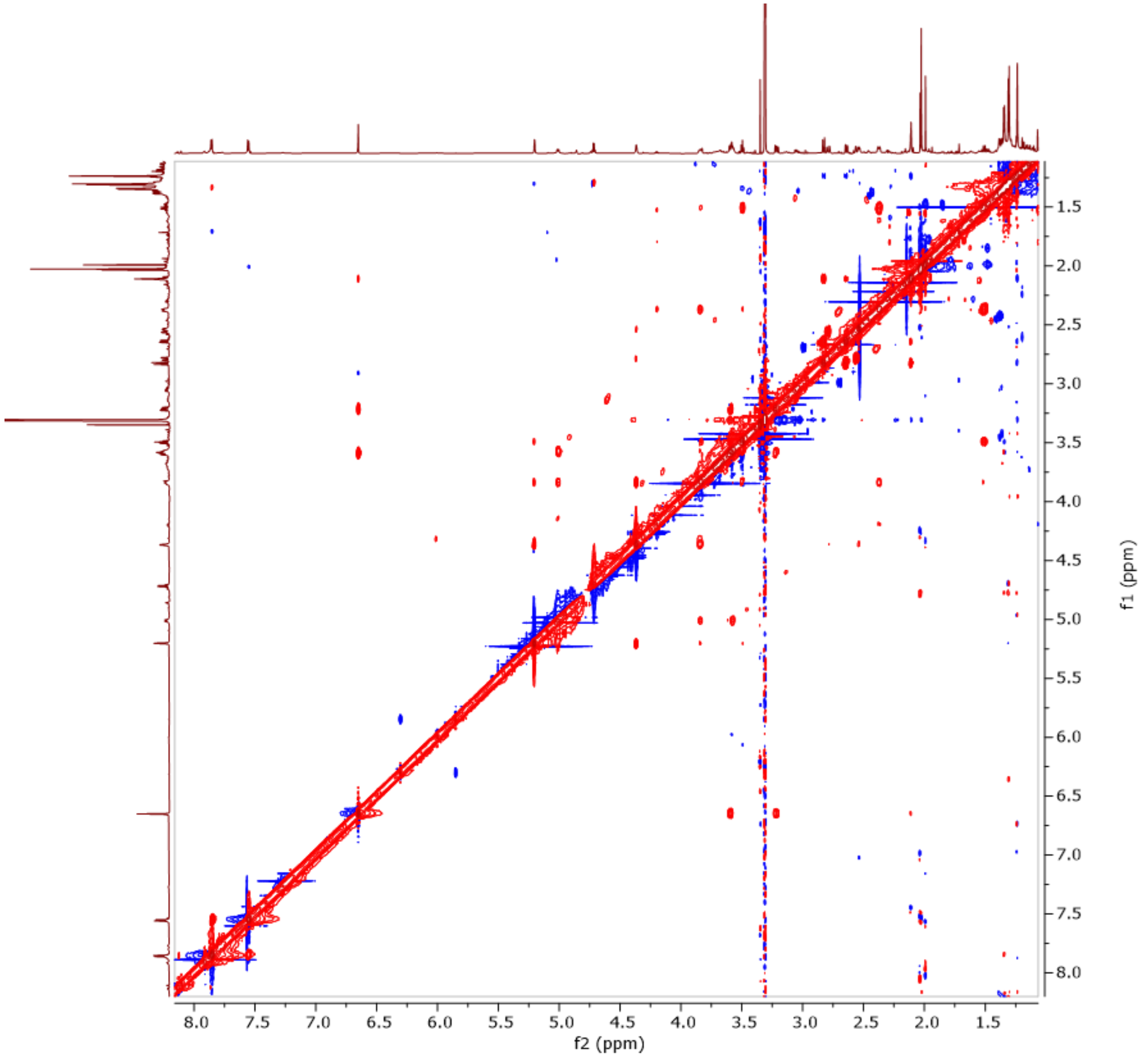


**Figure S15.** NOESY spectrum of saquayamycin N (**1**) (850 MHz, in $CD_3OD$).

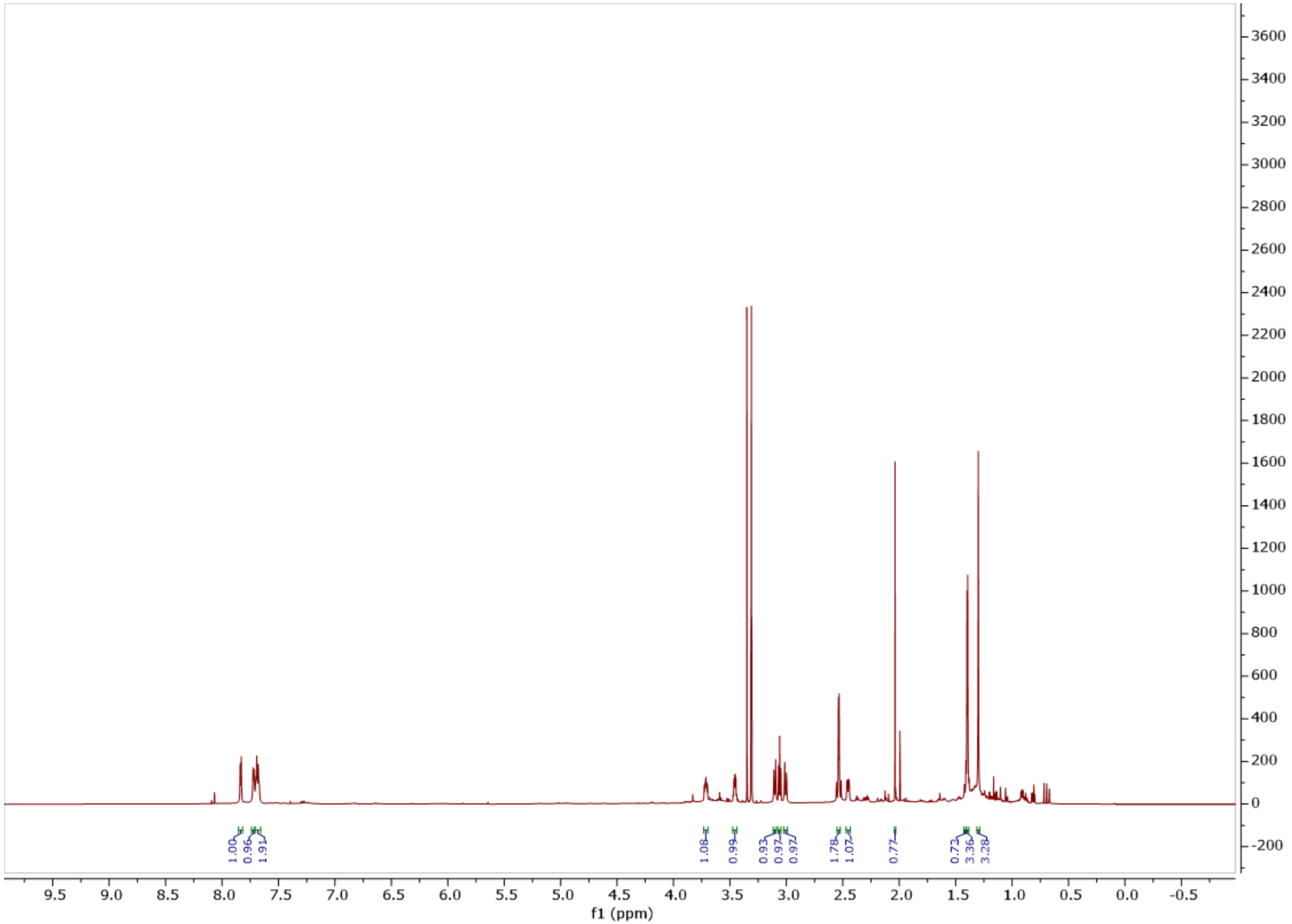


**Figure S16.** $^1$H NMR spectrum of fridamycin A (**2**) (850 MHz, in $CD_3OD$).

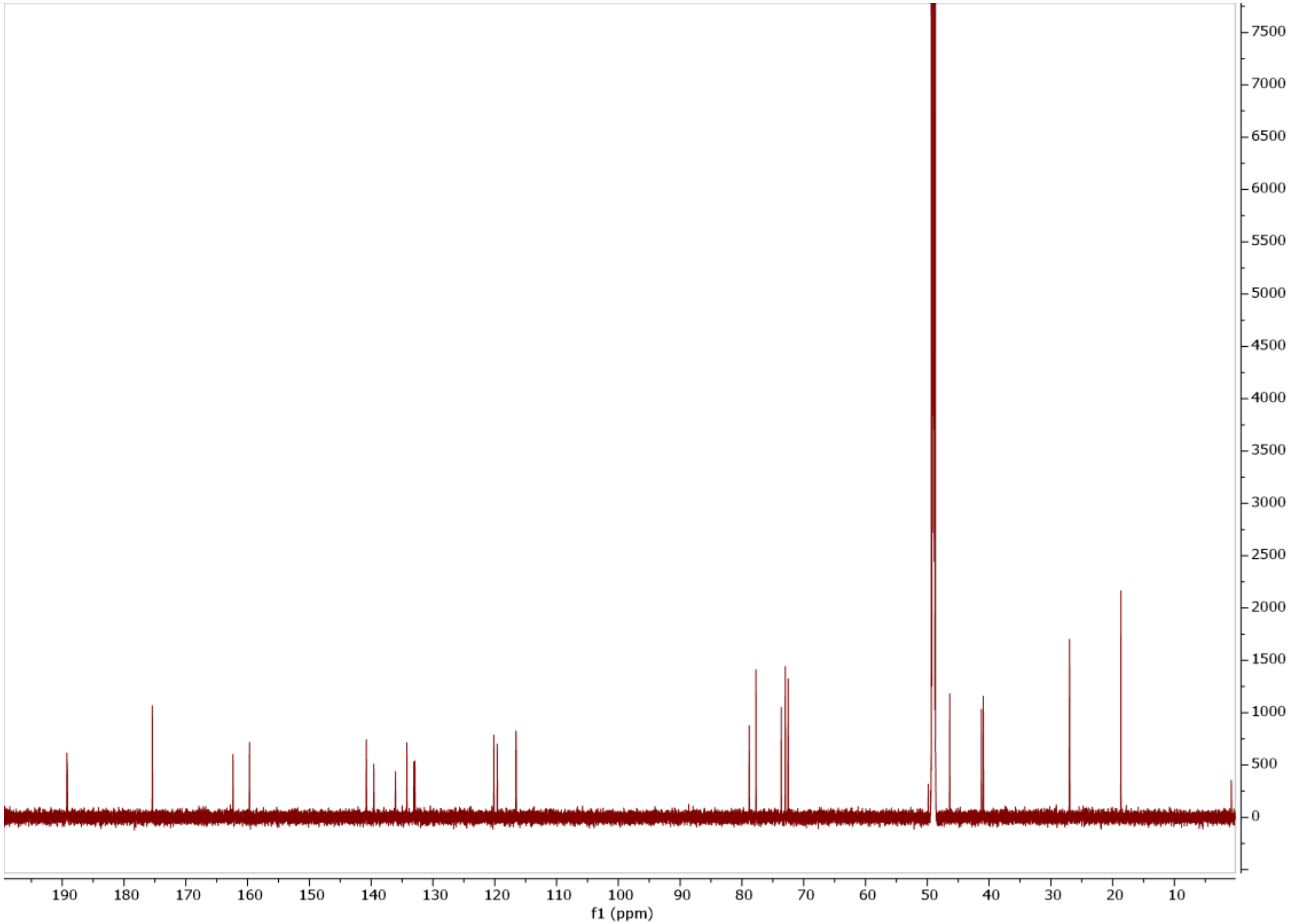


**Figure S17.** $^{13}$C NMR spectrum of fridamycin A (**2**) (213 MHz, in $CD_3OD$).

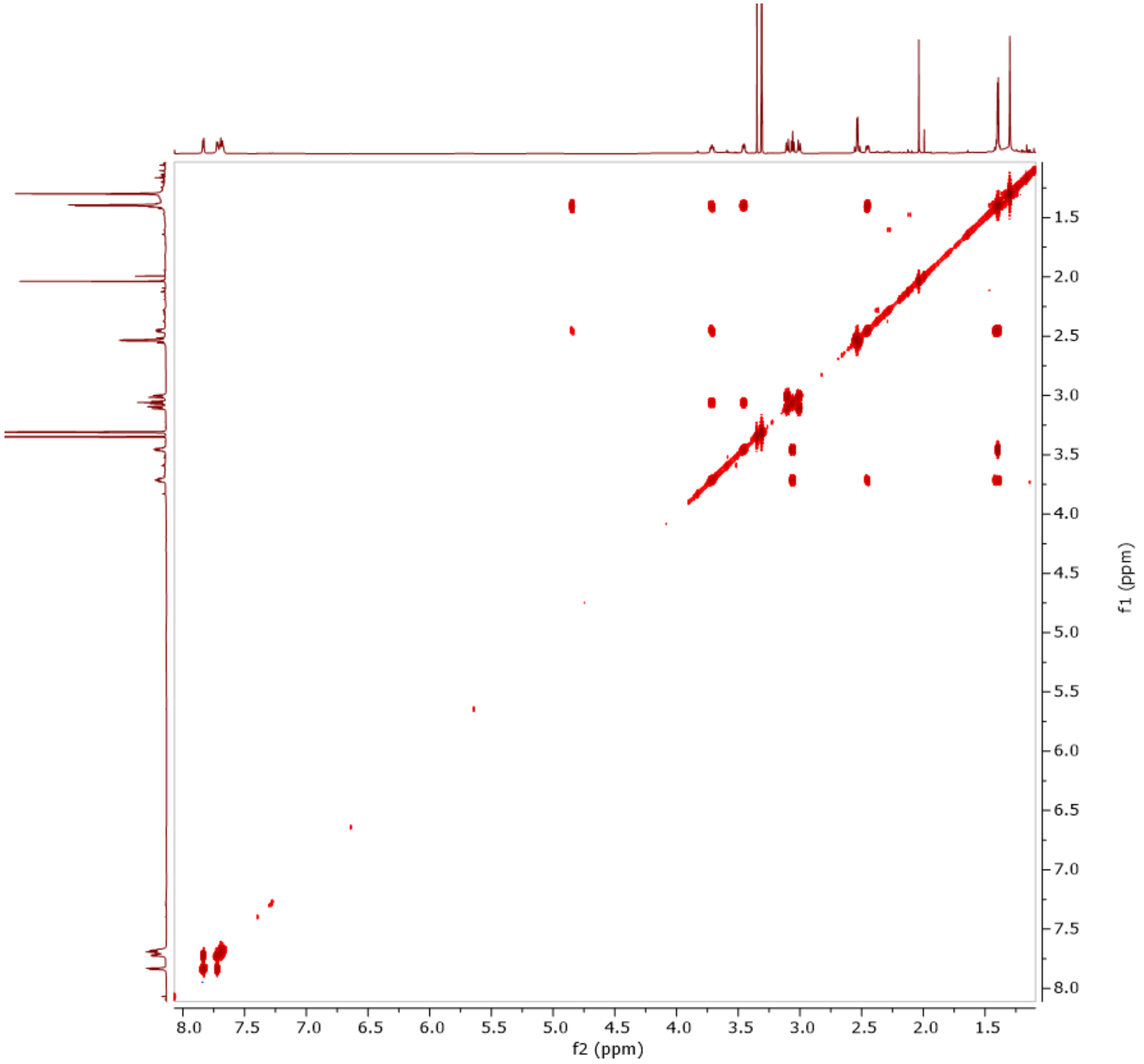


**Figure S18.** COSY spectrum of fridamycin A (**2**) (850 MHz, in $CD_3OD$).

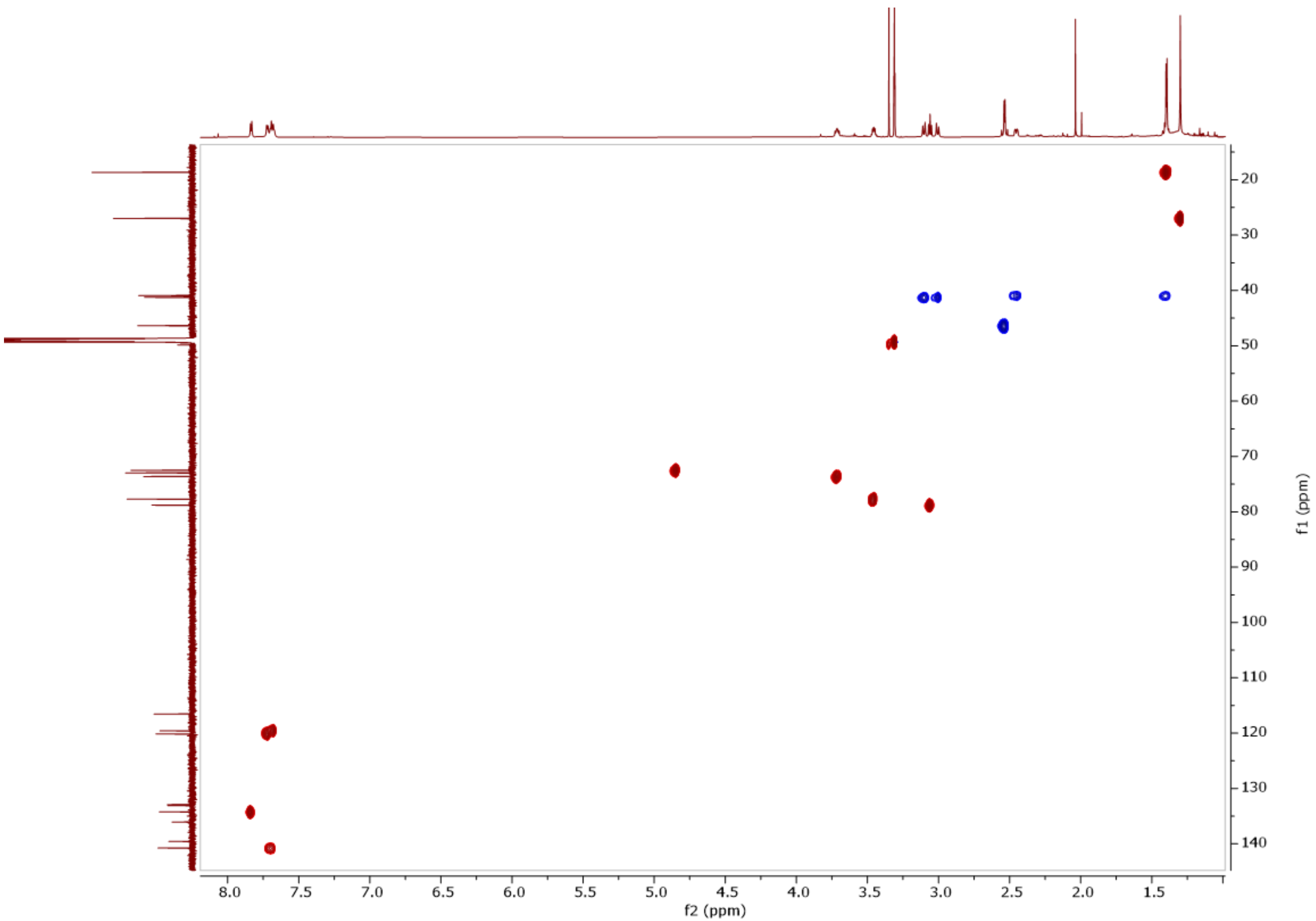


**Figure S19.** Multiplicity-edited HSQC spectrum of fridamycin A (**2**) (850 MHz, in $CD_3OD$).

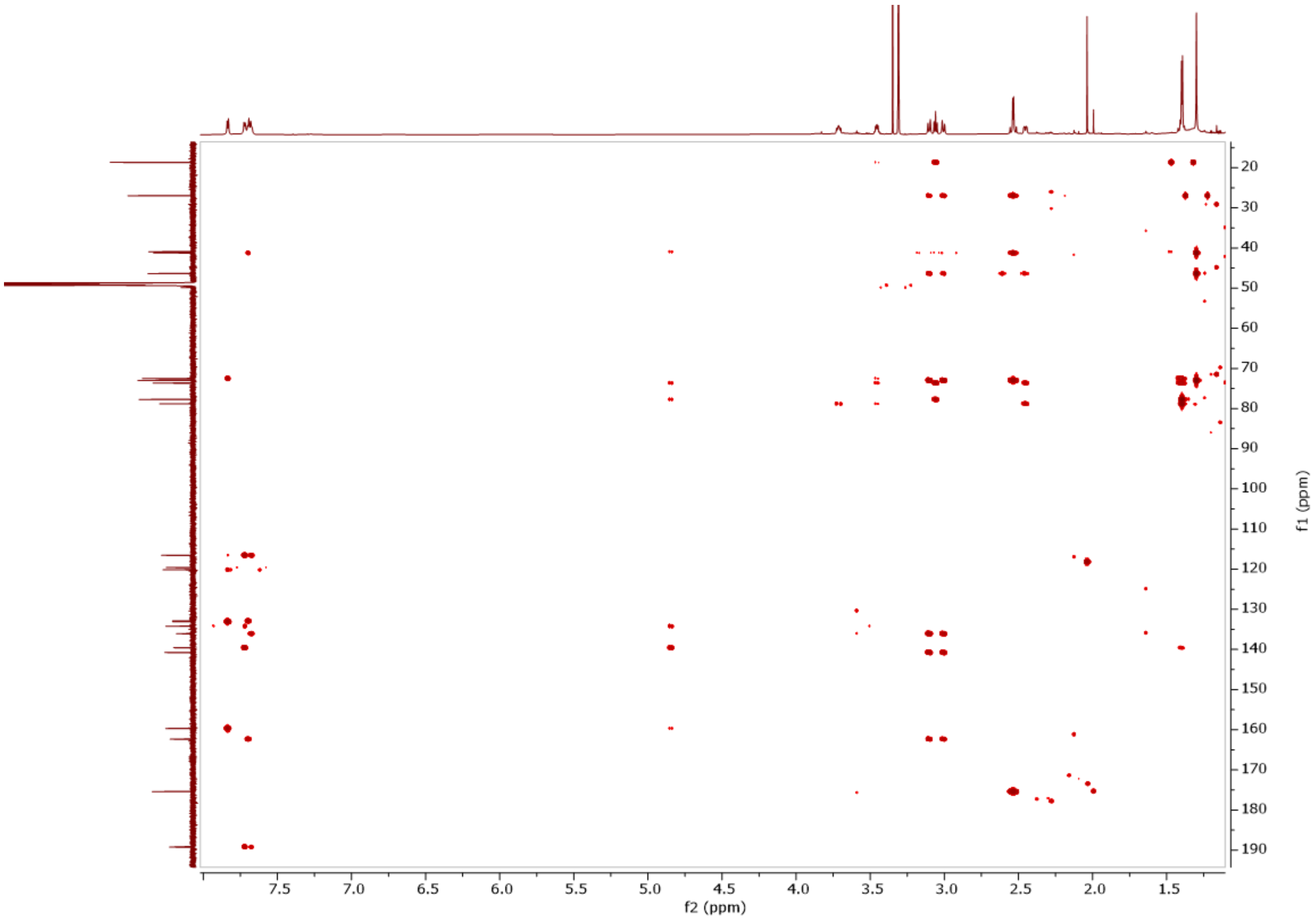


**Figure S20.** HMBC spectrum of fridamycin A (**2**) (850 MHz, in $CD_3OD$).

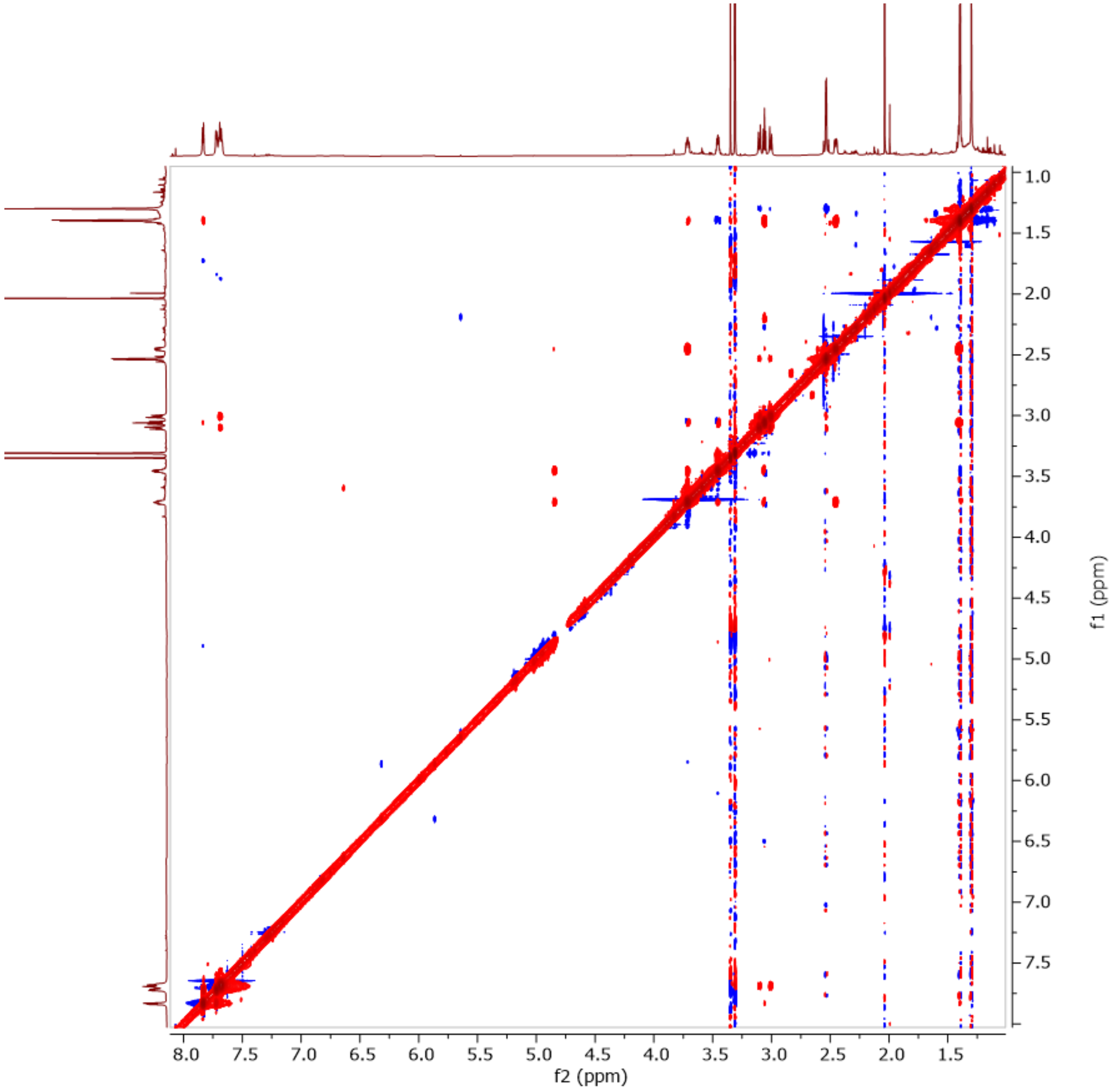


**Figure S21.** NOESY spectrum of 1 fridamycin A (**2**) (850 MHz, in $CD_3OD$).
